# Development of the mouse *anterior* amygdalar radial unit marked by *Lhx9*-expression

**DOI:** 10.1007/s00429-020-02201-8

**Published:** 2021-01-30

**Authors:** Elena Garcia-Calero, Luis Puelles

**Affiliations:** Department of Human Anatomy, School of Medicine and IMIB-Arrixaca Institute, University of Murcia, 30120 Murcia, Spain

**Keywords:** Radial amygdalar model, Pallium, Ventral pallium, Pallio-subpallial boundary, Pallial amygdala, Medial amygdala

## Abstract

The amygdala in mammals plays a key role in emotional processing and learning, being subdivided in pallial and subpallial derivatives. Recently, the cortical ring model and the pallial amygdalar radial model (Puelles et al. 2019; Garcia-Calero et al. 2020) described the pallial amygdala as an histogenetic field external to the allocortical ring, and subdivided it in five major radial domains called *lateral, basal, anterior, posterior and retroendopiriform* units. The* anterior* radial unit, whose cells typically express the *Lhx9* gene (see molecular profile in Garcia-Calero et al. 2020), is located next to the pallial/subpallial boundary. This radial domain shows massive radial translocation and accumulation of its derivatives into its intermediate and superficial strata, with only a glial palisade representing its final periventricular domain. To better understand the development of this singular radial domain, not described previously, we followed the expression of *Lhx9* during mouse amygdalar development in the context of the postulated radial subdivisions of the pallial amygdala and other telencephalic developmental features.

## Introduction

The telencephalic amygdala of mammals is a mixed pallial/subpallial nuclear complex located at the tip of the temporal lobe (Burdach 1819–1822; Johnston 1923; Loo 1930, 1931; De Olmos et al. 1985, 2004; Alheid et al. 1995; Swanson and Petrovich 1998; Martínez-García et al. 2012; Olucha-Bordonau et al. 2015, Medina et al. 2017). It is implicated in evaluation of combined external stimuli, processing of fear and other emotions, and consequent learning (Weiskranft 1956; Amaral et al. 2003; Phelps and Ledoux 2005; Ledoux, 2007; Rolls 2014, 2015). It contains a variety of nuclei and superficial corticoid structures with different pallial or subpallial embryonic origins, which are strongly interconnected (Johnston 1923; Krettek and Price 1978; Pitkänen et al. 1997; Smith-Fernandez et al. 1998; Puelles et al. 2000, 2016a, 2019; Medina et al. 2004; Tole et al. 2005; García-López et al. 2008; Hirata et al. 2009; Waclaw et al. 2010; Olucha-Bordonau et al. 2015; Medina et al. 2017; Desfilis et al. 2018). The subpallial amygdala includes the anterior, central and medial amygdalar nuclei, characterized by a high number of GABA-ergic cells (Swanson and Petrovich 1998). The pallial amygdala encompasses various amygdalar superficial corticoid masses (CxA, ACo, PLCo, PMCo) together with the classical basolateral/basomedial complex (lateral, basolateral and basomedial nuclei) and the amygdalo-hippocampal area (AHi) (Swanson and Petrovich 1998; Puelles et al. 2000, 2016a; Medina et al. 2004, 2017; Hirata et al. 2009; Waclaw et al. 2010). Corticoid and nuclear pallial amygdalar areas are often studied as separate entities.

A number of modern developmental studies which attended to the amygdalar molecular profile contributed significantly to our present partial understanding of pallial amygdalar patterning and progenitor sources (e.g., Smith-Fernandez et al. 1998; Puelles et al. 2000, 2016a; Medina et al. 2004, 2017; Remedios et al. 2004, 2007; Tole et al. 2005; Bielle et al. 2005; García-López et al. 2008; Hirata et al. 2009; Waclaw et al. 2010; Ruiz-Reig et al. 2018; Garcia-Calero et al. 2020). For instance, expression of the *Emx1* transcription factor and other regulatory genes suggested a subdivision of the pallial amygdala into molecularly distinct ventral pallial and lateral pallial territories (Smith-Fernández et al. 1998; Puelles et al. 2000; Gorski et al. 2002; Medina et al. 2004; Cocas et al. 2011; Martínez-García et al. 2008, 2012; Olucha-Bordonau et al. 2015). In addition, Remedios et al. (2007) conjectured that the ectopically migrated amygdalar nucleus of the lateral olfactory tract (NLOT) originates from a caudal part of the *dorsal pallium*. Other studies proposed a common embryonic source for basolateral and corticoid amygdalar structures in the *ventral pallium* (Stenman et al. 2003; Hirata et al. 2009; Waclaw et al. 2010). *Dbx1* was considered a selective marker of ventropallial progenitors in the telencephalic pallium (Yun et al. 2001; Medina et al, 2004; Bielle et al. 2005; Puelles et al. 2016a). Study of *Dbx1*-derived lineage suggested that the *ventral pallium* was only a *partial* source of excitatory neurons populating the basolateral amygdalar nuclear complex and the cortical amygdala (Hirata et al. 2009; Waclaw et al. 2010). Indeed, Puelles et al. (2016a) mapped *Dbx1*-LacZ-labelled neuronal derivatives, and identified the *Dbx1*-positive *ventral pallium* as the origin of anterior parts of the basolateral amygdalar complex. The observation that some caudal parts of the same complex were apparently largely devoid of *Dbx1*-derived cell populations was not easy to explain. It was suggested as one possibility that a dorsal part of the *ventral pallium* progenitors might not express this marker (thus leading to lack of LacZ signal in the corresponding neuronal derivatives; this notion was proposed by LP in Puelles et al. 2016a). Another possible explanation adduced was that there might exist an extra amygdalar pallial subdivision, i.e., not ventropallial in nature, which was devoid of *Dbx1*-positive progenitors. Such hypothetic non-ventropallial domain, which would occupy the caudal amygdala, proposed originally by L.Medina, was tentatively identified as a *ventrolateral caudal pallium* (or *ventrocaudal pallium*) (Puelles et al. 2016a; see also Abellán et al. 2014; Medina et al. 2017; Desfilis et al. 2018). Ruiz-Reig et al. (2018) defined an apparently different ‘caudoventral’ amygdalar sector, which reportedly contributes cells to the subpallial medial amygdala.

On the other hand, Puelles (2014, 2017) and Puelles et al. (2016b) analysed critically the classic notion of a claustroamygdalar complex (Kuhlenbeck 1924, 1927; Holmgren 1925), which had been assumed to exist by Puelles et al. (2000) and Medina et al. (2004). The selective claustral marker *Nr4a2* was examined throughout development in the mouse, concluding that the resulting updated *lateral pallium* sector (represented by a *claustro-insular* radial histogenetic complex; see also Puelles et al. 2019) does not reach the amygdalar domain. According to these new data, the pallial amygdala has no part of *lateral pallium*, contrarily to what was concluded by Medina et al. (2004).

Finally, Abellán et al. (2014) ascribed the periventricular AHi area and the posteromedial corticoid nucleus (PMCo) to the *medial pallium* on the basis of a number of shared molecular markers. This brief abstract of relevant literature shows that distinct *ventral*, *ventrolateral caudal*, *caudoventral*, *lateral*, *dorsal* and *medial* pallial portions have been ascribed to the pallial amygdala, though some of the early proposals have been subsequently negated (case of *lateral pallium*), qualified (case of *ventral pallium*), or doubted (case of *dorsal pallium*; see below). The new *ventrolateral caudal* and *caudoventral* subdivisions are still being tested.

Recently, other approaches threw new light on this difficult topic. First, Puelles et al. (2019) and Garcia-Calero et al. (2020) deduced from the concentric ring model of the telencephalic cortical pallium and correlative molecular mappings that the pallial amygdala is wholly external in nature to the whole cortex-like pallium and thus should be considered histogenetically independent as a separate *amygdalar pallium field* (a point that was unclear before; most authors tended to assume that amygdalar populations were produced within given cortical pallial sectors and thereafter migrated tangentially to their final amygdalar sites, though such migrations were not demonstrated; see Deussing and Wurst 2007). Puelles et al. (2019) and Garcia-Calero et al. (2021) expressed doubts about a *dorsopallial* origin of the amygdalar NLOT nucleus (postulated by Remedios et al. 2007) due to inconsistency of this notion within the concentric ring model (i.e., there is no dorsal pallium next to the separate amygdalar pallial neighbourhood). Secondly, we recently examined the radial (glial) dimension of the mouse pallial amygdala, aiming to identify its intrinsic radial histogenetic subdivisions, and clarify the ascription of individual amygdalar nuclei relative to periventricular, intermediate or superficial strata within these units (Garcia-Calero et al. 2020). Our results revealed five major radial units and some radial subdivisions side by side, totalling nine radial modules (see summary of radial amygdalar units, subunits and derived nuclei in Table1; a 3D schema representing these amygdalar units is found in Garcia-Calero et al. 2020 and Garcia-Calero and Puelles 2020).

**Table 1:** Amygdalar pallial nuclei distributed in periventricular, mantle and superficial strata in the amygdalar radial units (*lateral*, *basal*, *anterior*, *posterior* and *retroendopiriform*) or subunits (for *basal* and *posterior* units). For abbreviations, see list

Units	Lateral	Basal			Anterior	Posterior			Retroendopiriform
Subunits		Baso-lateral	Basomedio-lateral	Basomedio-medial		Rostro-lateral	Rostro-medial	Caudo-lateral	
Periventricular stratum	L	BLP	BMPL	BMPM	Glial palisade	AHiRL	AHiRM	AHiCL	REP
Intermediate stratum	LI	BLA/BLI	BMIL	BMIM	BMA	PMCoRLi	PMCoRMi	PMCoCLi	REPI
Superficial stratum	CxAR	CxAC	PLCoC	PLCoR	ACo	PMCoRLs	PMCoRMs	PMCoCLs	REPCo

The new status of the entire pallial amygdala field as topologically *external* to the telencephalic cortex (i.e., producing all its nuclei, rather than receiving them via migrations, irrespective of its contact with some cortical areas and the existence of shared gene markers) draws new attention to amygdalar borders (Puelles et al. 2019). It was proposed that the pallial amygdala lies intercalated between the alar hypothalamus, ventrally, and caudo-ventral parts of the outer allocortical ring, dorsally. These include the continuum formed by the caudal periamygdalar piriform cortex, with its amygdalo-piriform specialized area, the entorhinal schizocortex and the hippocampal complex. The amygdalar pallial field in addition contacts the subpallium (its striatal, pallidal and diagonal domains, where central, medial and anterior amygdala subpallial domains arise; Puelles et al. 2013, 2016b; Garcia-Calero et al. 2020). The pallial amygdala also contacts caudally the prethalamic eminence (rostrodorsal diencephalon; Puelles et al. 2019; Alonso et al. 2020). These multiple planar relationships still need to be examined in more detail to assess their relevance for causal developmental explanation of amygdalar pattern.

The present report is centred on the *anterior* radial unit, which was first defined in our radial model of the pallial amygdala. It lies next to the pallio-subpallial border and thus might relate to cortical ventral pallium represented in the neighbouring olfactory cortex (i.e., as suggested by *Dbx1-*LacZ-labelled progeny; Puelles et al. 2016a). Garcia-Calero et al. (2020) already concluded from selected developmental data and other results from the literature that this pallial amygdalar subdivision uniquely displays accumulation of its neuronal derivatives at its intermediate and superficial strata, developing thus a depopulated periventricular zone. This does not occur in the other eight pallial amygdalar radial modules, which retain periventricular derivatives. The intermediate and superficial components of this *anterior* radial domain (formed by the conventional anterior basomedial nucleus, BMA, and the anterior corticoid nucleus, ACo, respectively) separate the subpallium from the neighbouring *lateral* and *basal* radial units. Radial glia DiI-labelling experiments indicated that the exclusively glial periventricular zone of the *anterior* radial unit is next to the lateral (L) and the posterior basolateral (BLP) nuclei, laterally, and the pallio-subpallial boundary, medially, thus maintaining an equivalent border-related topologic position (Garcia-Calero et al. 2020). Analysis of some 80 amygdalar gene expression patterns mined from the Allen Developing Mouse Brain Atlas database (http://www.developingmouse.brain-map.org) demonstrated that 14 among the 20 genes found to be expressed in this unit (70%) distinguish the *anterior* radial domain from the other pallial amygdalar units (Garcia-Calero et al. 2020). The transcription factor gene *Lhx9* employed in the present work emerged as one of the selective markers for the whole *anterior* radial unit.

*Lhx9* is a Lim-homeodomain gene which is expressed in the telencephalon of vertebrates during development (Retaux et al. 1999; Bachy et al. 2001; Moreno et al. 2004; Tole et al. 2005; García-López et al. 2008; Abellán et al. 2009, 2013; Medina et al. 2017; Desfilis et al. 2018). Several analyses of the *Lhx9* expression pattern were published in recent years in mouse and chicken telencephalon, reportedly showing some overlap with the expression of its paralog *Lhx2* in the BMA and ACo nuclei, plus some other more caudal nuclei (Remedios et al. 2004; Tole et al. 2005; García-López et al. 2008; Abellán et al. 2009, 2010, 2013; Medina et al. 2017; Garcia-Calero et al. 2020). *Lhx9* expression was generally described by these authors as marking in general the *ventral pallium*; interestingly, its early radially distributed signal in the amygdala coincides topographically with a *Tbr1*-poor area found next to the subpallium (Tole et al. 2005). However, its distribution does not seem to coincide with that of *Dbx1-*LacZ-labelled progeny, supposed to represent the *ventral pallium* mantle (Puelles et al. 2016a). *Lhx9* gene transcripts were also observed in *subpallial* amygdalar regions such as the anterior amygdala (AA), parts of the medial amygdala, and the bed nucleus of the accessory olfactory bulb (BAOT) (García-López et al. 2008; Abellán et al. 2009, 2013; Medina et al. 2017; Garcia-Calero et al. 2020).

In the present work, we studied in more detail the developmental expression of *Lhx9* as a marker of the *anterior* amygdalar radial unit, and eventually of other amygdalar nuclei. We compared the expression of this gene with other significant cortical and amygdalar gene markers such as the Tbr1 protein (a general pallial marker; Puelles et al. 2000; Hevner et al. 2001; Medina et al. 2004), the subpallial marker *Dlx5* (Puelles et al. 2000; Medina et al. 2004; Cobos et al. 2006), the Lim-homeodomain gene *Lhx2* (Bulchand et al. 2003; Tole et al. 2005), and the *kelch* family gene *Enc1* (Garcia-Calero and Puelles 2005; Garcia-Calero 2005).

Our early results at E12.5 corroborate the previously described complete ventriculo-pial radial distribution of the *Lhx9*-labelled domain, identified by us as the primordium of the *anterior* amygdalar radial unit, whereas no such signal was found at the neighbouring *lateral* and *basal* radial units, which contrasted by expressing instead selectively the *Enc1* gene marker. This already raised questions about the apparent heterogeneity of amygdalar components thought to be ventropallial (Puelles et al. 2016a). Subsequently, at mid- and late-embryonic, or perinatal, stages, there is a progressive decrease in the number of *Lhx9* positive cells found in periventricular and deep intermediate strata of the *anterior* radial amygdalar unit, accompanied by clear-cut accumulation of *Lhx9* transcripts at correlative superficial intermediate and cortical *anterior* strata (BMA and ACo nuclei). Moreover, other *Lhx9-*positive amygdalar formations gradually emerge at the caudomedial amygdalar pole. The *posterior* radial unit (AHi/PMCo), held to be a derivative of the *medial pallium* (see above) becomes strongly labelled, as well as the likewise caudal (and novel) *retroendopiriform* radial unit (Garcia-Calero et al. 2020; Table 1). Both are spatially separated from the *anterior* radial unit.

The present results, together with correlative *Enc1* and Tbr1 data, plus a re-evaluation of the *Dbx1*-derived progeny data of Puelles et al. (2016a), corroborate the molecular singularity of the mode of development of each of the *diverse* amygdalar radial units (Garcia-Calero et al. 2020), all of which seem in retrospect to derive from *Dbx1*-positive neuroepithelium. This leads us to discuss the issue whether it is helpful to extrapolate cortical pallial sectors into the separate pallial amygdalar field, concluding that it may be advantageous not to do so. We also discuss minimally the apparent functional role of the anterior amygdalar unit within the amygdalar system.

## Results

### *Lhx9* expression in the amygdalar domain at early developmental stages

**Fig. 1:**
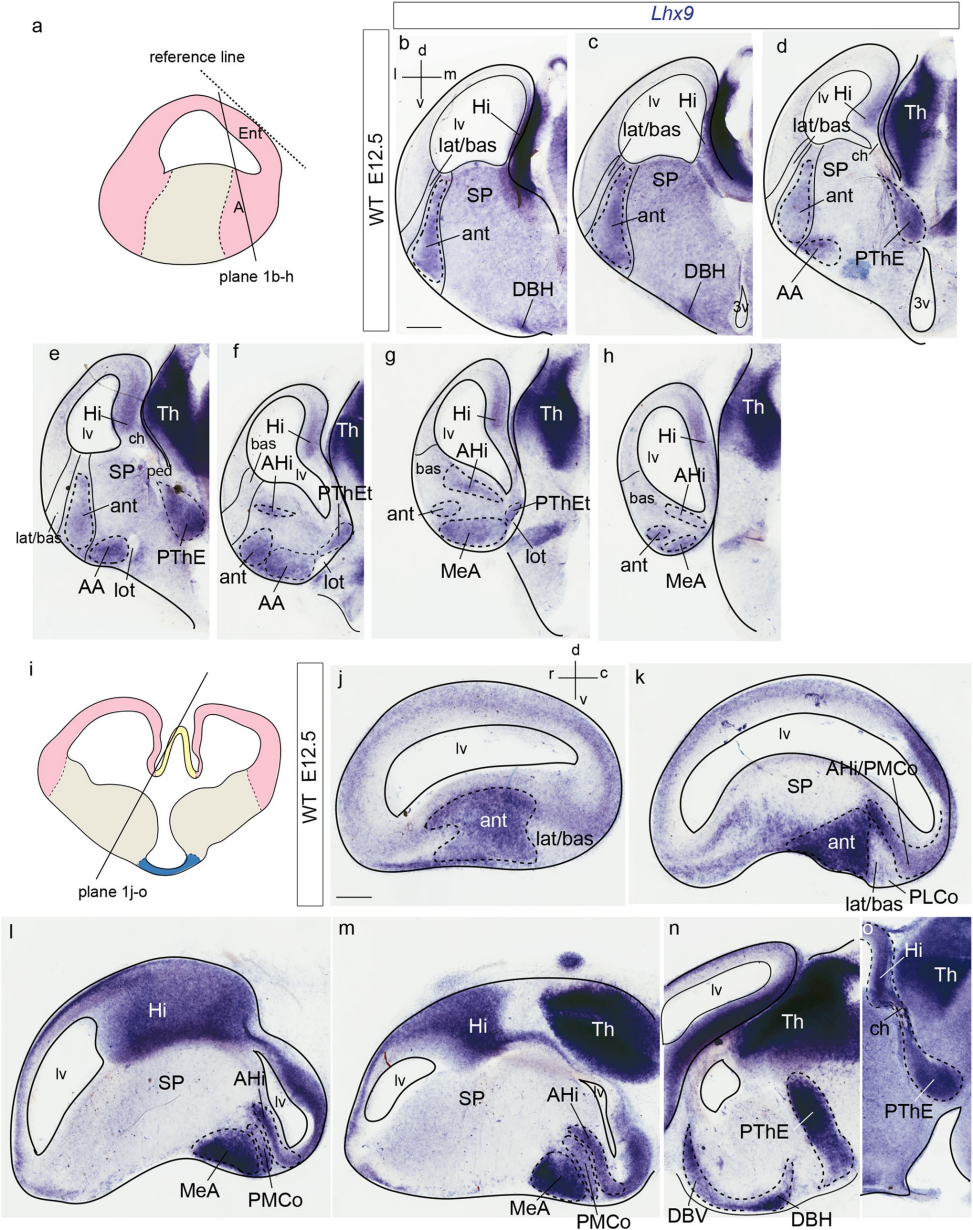
Amygdalar *Lhx9* expression in stage E12.5 mouse embryos. **a**, **i** Schematic representation of section planes: **a** amygdalar radial plane for figures (**b**–**h**); **i** oblique sagittal plane for figures (**j**–**o**). **b**–**h** *Lhx9* expression images ordered from rostral to caudal levels; **b** orientation in upper left-hand corner. The limits between the *lateral/basal complex* and the olfactory cortex, as well as between the *lateral/basal* complex and the *anterior* radial unit, or the latter and subpallium are indicated with thin black lines. **j**–**o** Sagittal *Lhx9* expression images ordered from lateral to medial levels; **j** orientation in upper right-hand corner. For abbreviations, see list. Scale bars in **b**–**o** represent 300 µm

**Fig. 2:**
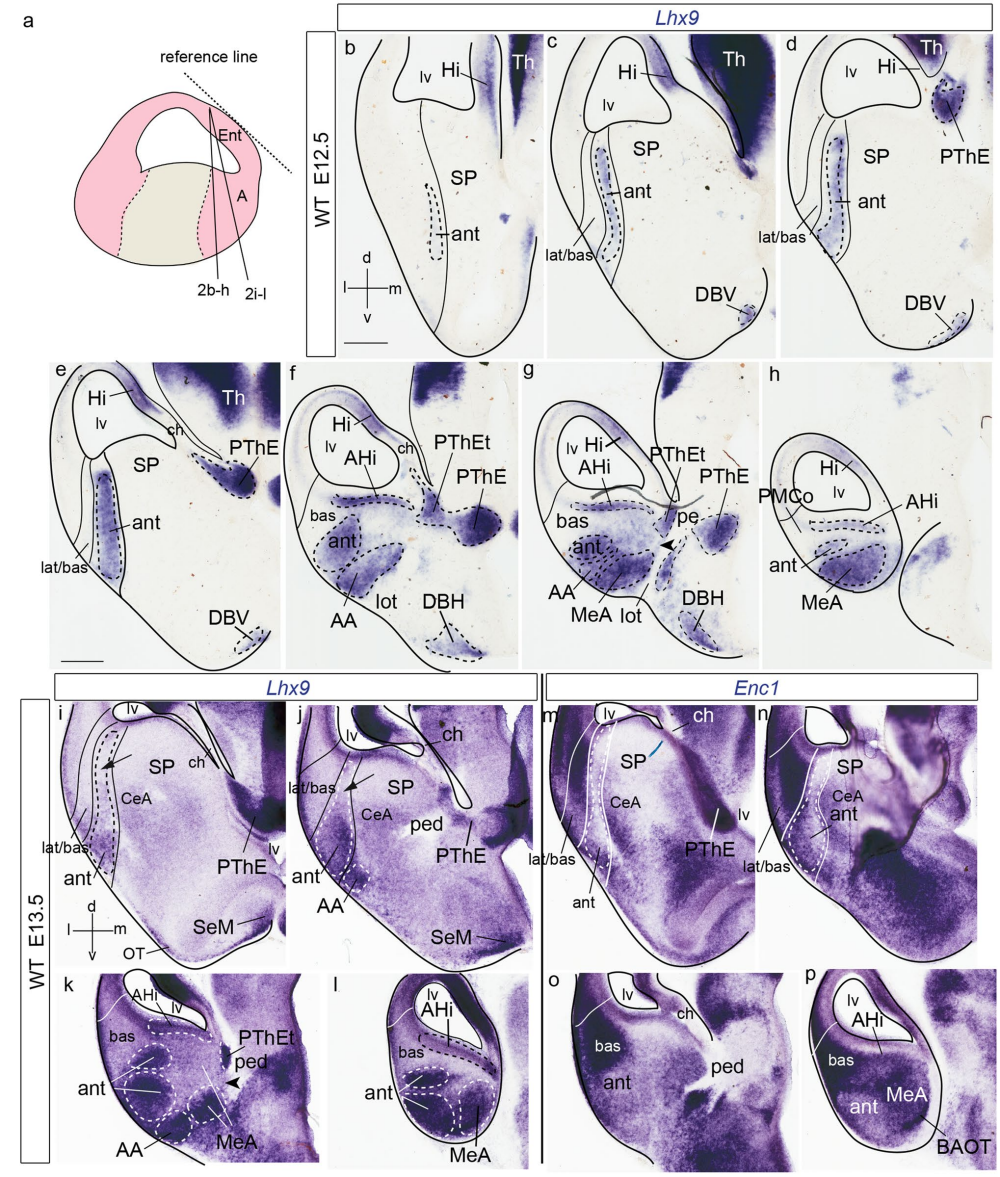
*Lhx9* and *Enc1* expression in mouse amygdalar region at embryonic stages E12.5 and E13.5. **a** Schematic representation of section plane. **b**–**h** *Lhx9* expression at stage E12.5 ordered from rostral to caudal levels; **b** orientations indicated at the bottom left-hand corner. **i**–**l** *Lhx9* expression at stage E13.5 in a slightly different amygdalar radial plane (see **a**), ordered from rostral to caudal levels. **m**–**p** *Enc1* expression at stage E13.5, ordered from rostral to caudal levels, in alternate sections from the same embryo as in (**i**–**l**); **i**, **j** black arrow indicates a *Lhx9*-negative gap in the deep strata of the *anterior* amygdalar radial unit; orientation indicated in the bottom left-hand corner of (**i**). **g**, **k** black arrowhead point to positive *Lhx9* cells in the medial amygdalar surface with possible source in PThEt. The limits between the lateral/basal complex and olfactory cortex, between the* lateral/basal* complex and the* anterior* radial unit, or the latter and subpallium are indicated with black or white lines. For abbreviations, see list. Scale bars represent 300 µm (**b**–**h**) and 350 µm (**i**–**p**)

We examined the changes in *Lhx9* expression during amygdalar development (Figs. 1, 2) within the conceptual context of our recently proposed *radial model of the pallial amygdala*, wherein 5 amygdalar radial units were defined (Table 1; Garcia-Calero et al. 2020). Summary reference to late-embryonic *Lhx9* expression was already made in that report; present results explore this aspect in more depth. Apart standard section planes, we also used the *amygdalar radial plane* (*loc.cit*.), which fits optimally the radial disposition of amygdalar glial bundles (the section planes used were indicated in the figures). Briefly, the *amygdalar radial section plane* intersects jointly the ventricle and the pial surface of the pallial amygdala with an obliquity of 30–45 degrees relative to conventional coronal sections; this angle was calculated while embedding the brain, by orienting the block’s basis and cutting surface relative to a reference plane tangential to the relatively flat entorhinal cortex; see Fig. 1 in Garcia-Calero et al. 2020). Comparison of *Lhx9* signal with *Enc1* transcripts helped us understand the disposition of the *Lhx9*-positive domain within the amygdala (Fig. 2) (Garcia-Calero and Puelles 2005; García-Calero 2005).

At E12.5, *Lhx9* expression adopts a full radial distribution in the amygdalar pallial region, with transcripts extending uniformly from periventricular levels to the brain surface, always next to the pallio-subpallial boundary (ant; Figs. 1b–h, j, k; 2b–h). The *lateral* and *basal* radial units are respectively disposed lateral and latero-caudal to the *anterior* unit, and do not show *Lhx9* expression (lat/bas in the same images). The subpallium is largely *Lhx9*-negative, with exception of some *Lhx9-*positive cells which spread subpially out of the *anterior* unit into the anterior amygdala, and positive cells observed at the vertical and horizontal nuclei of the diagonal band (lat/bas; bas; ant; SP; AA; DBV and DBH; Figs.1b–f, j-l, n; 2c–g; note PLCo is the superficial structure of the *basal* amygdalar unit; see also Fig. 2i–p). At caudal superficial levels, there is also an expansion of *Lhx9*-positive cells that invades parts of the prospective medial amygdala (probably by tangential migration; Bupesh et al. 2011; Garcia-Calero et al. 2020). There is separate labelling of the *posterior* radial unit, mainly at its periventricular zone, the amygdalo-hippocampal area (AHi; MeA; Figs.1f–h, k-m; 2f–h). On the whole, there is abundant expression of *Lhx9* at the amygdalar caudal pole, presided by the superficial ACo structure of the *anterior* radial unit. It is note-worthy that the *Lhx9*-positive BMA lacks contact with the periventricular or ventricular zones and displays a rounded form (Figs. 1f–h; 2f–h). The caudal *Lhx9* expression separately limits medially with the caudal end of the lateral olfactory tract, an area where the *Lhx9*-positive BAOT nucleus will later become distinct (lot; Figs. 1e-g; 2f, g; Garcia-Calero et al. 2020; their Figs. 8d, e).

A separate forebrain region in this area which also expresses *Lhx9* is the prethalamic eminence (PThE). This shows at E12.5 intense *Lhx9* signal (PThE; or its telencephalic subarea PThEt; Figs. 1d–g, n, o; 2d–g). The *Lhx9*-positive PThE has its topologically dorsal end at the fissural chorioidal tela (a derivative of the roof plate), which attaches on its other side to the hippocampal fimbrial hem (also a *Lhx9* positive area) (PThE; PThEt; ch; Hi; Figs. 1c–e, m, o; 2d–f). No contact was observed between the chorioidal tela and the amygdala proper.

At E13.5, amygdalar *Lhx9* expression again labels the *anterior* radial domain next to the pallio-subpallial boundary, as in the previous stage (ant; Fig. 2i–l). However, there is hardly any *Lhx9* signal at the *anterior* periventricular stratum, thus creating a clear-cut separation between the still weakly *Lhx9*-positive *anterior* ventricular stratum and the corresponding, more strongly positive intermediate and superficial strata of the *anterior* mantle (arrow; Fig. 2i, j). We compared this pattern with amygdalar *Enc1* expression in adjacent sections, which identifies the *lateral/basal* complex (Garcia-Calero et al. 2020). Strong *Enc1* signal at this complex extends fully radially to its superficial component, the rostral amygdalo-cortical transition area (lat/bas; Fig. 2m, n). The *lateral/basal* complex appears clearly delimited medially by an *Enc1*-negative gap which separates that complex from the central amygdalar nucleus, a part of the striatal subpallium shows weak *Enc1* expression (CeA; Fig. 2m, n). This unlabelled gap is the site of the increasingly depopulated deep intermediate and periventricular *anterior* unit, already much reduced in width at this stage (ant; lat/bas; bas; CeA; Figs. 2m–p). The *Lhx9*-positive intermediate and superficial *anterior* strata are instead wider than at E12.5, and show superficial contiguity with a larger mass of positive elements apparently invading the anterior amygdala, and maybe even the olfactory tuberculum (OT; AA; Fig. 2i, j). At more caudal levels, the *Enc1* signal at the *lateral/basal* complex starts to expand medialwards, but is still separated at this locus from the* posterior* unit (AHi) by a deep *Lhx9*-positive portion of the *anterior* unit mantle; the latter seems here less separated from the corresponding ventricular zone than more rostrally (bas; ant; AHi; Figs. 2o, p). A definite separation is nevertheless observed slightly more caudally between the *Enc1*-positive *basal* complex, which has finally become continuous medially with the *posterior* unit (AHi), and the caudal end of the *anterior* unit (lat/bas; ant; AHi; Fig. 2l, p). The medial amygdala and AHi also show *Lhx9* signal, consistent with that observed already at E12.5 (MeA; AHi; Fig. 2k, l). The stretched telencephalic endpart of the *Lhx9* positive PThE domain, now seen separated from the main non-telencephalic PThE by the incipient cerebral peduncle, can be distinguished at this stage as a small *Lhx9*-positive patch at the medial telencephalic surface, next to the chorioidal fissure. The PThEt seems tenuously in contact with *Lhx9-*positive cells in the MeA possibly including cells spread over the lot (PThEt; MeA; Fig. 2k, l; arrowhead; Figs. 1g, 2k).

### *Lhx9* transcripts at intermediate and perinatal stages

**Fig. 3:**
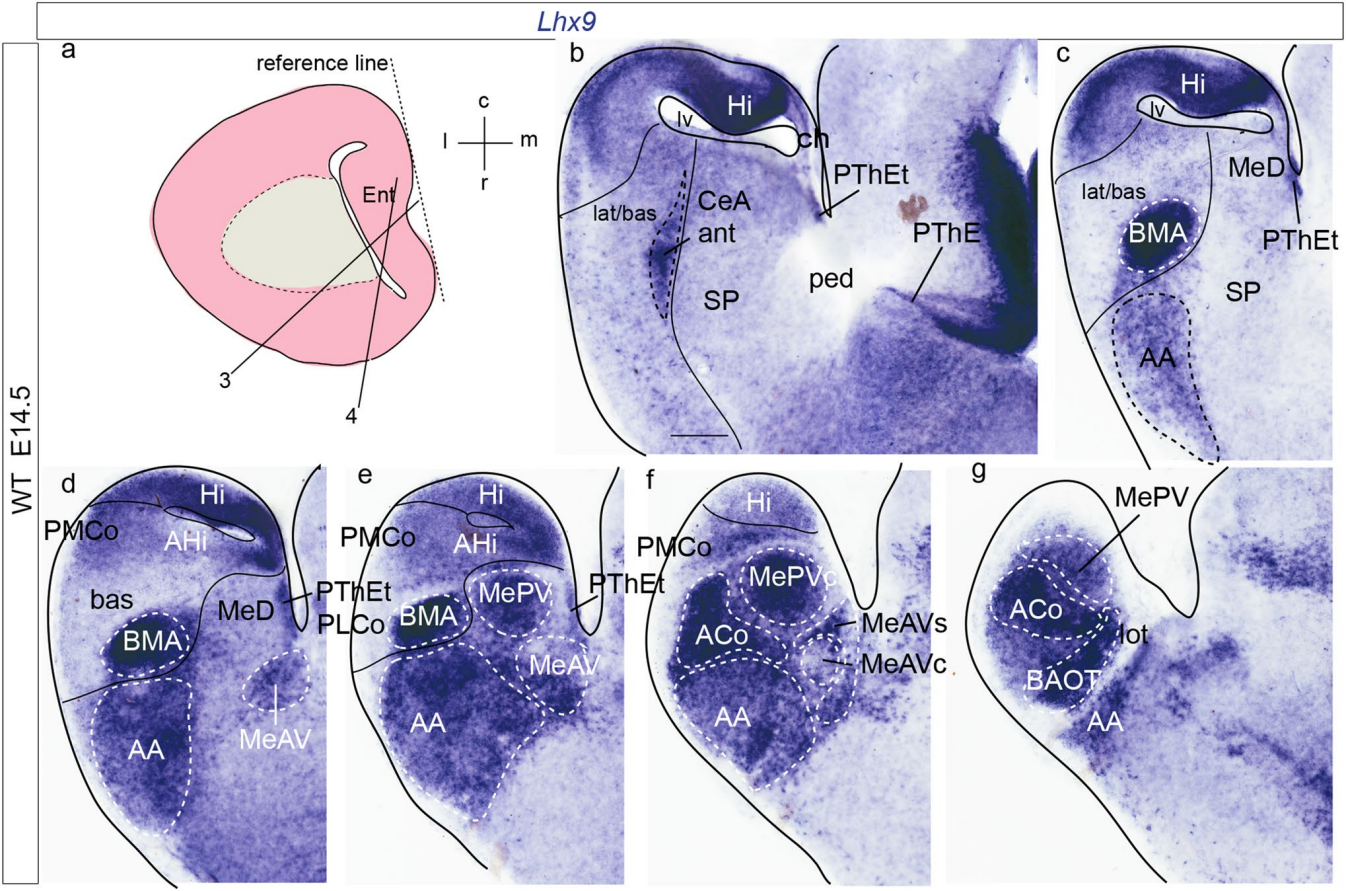
*Lhx9* expression in mouse amygdalar region at embryonic stage E14.5, in nearly horizontal sections ordered from dorsal to ventral levels. Note lack of continuity of *anterior* unit signal with the ventricle. **a** Schematic representation of section plane for Figs.3 and 4; **b** orientation indicated in the upper right-hand corner. The limits between the *posterior* radial unit and the hippocampus, the *lateral/basal* complex and the cortex, as well as between the *lateral/basal* complex and the *anterior* radial unit, or the latter versus the subpallium are indicated with black lines. For abbreviations, see list. Scale bar represent 250 µm (**b**–**g**)

**Fig. 4:**
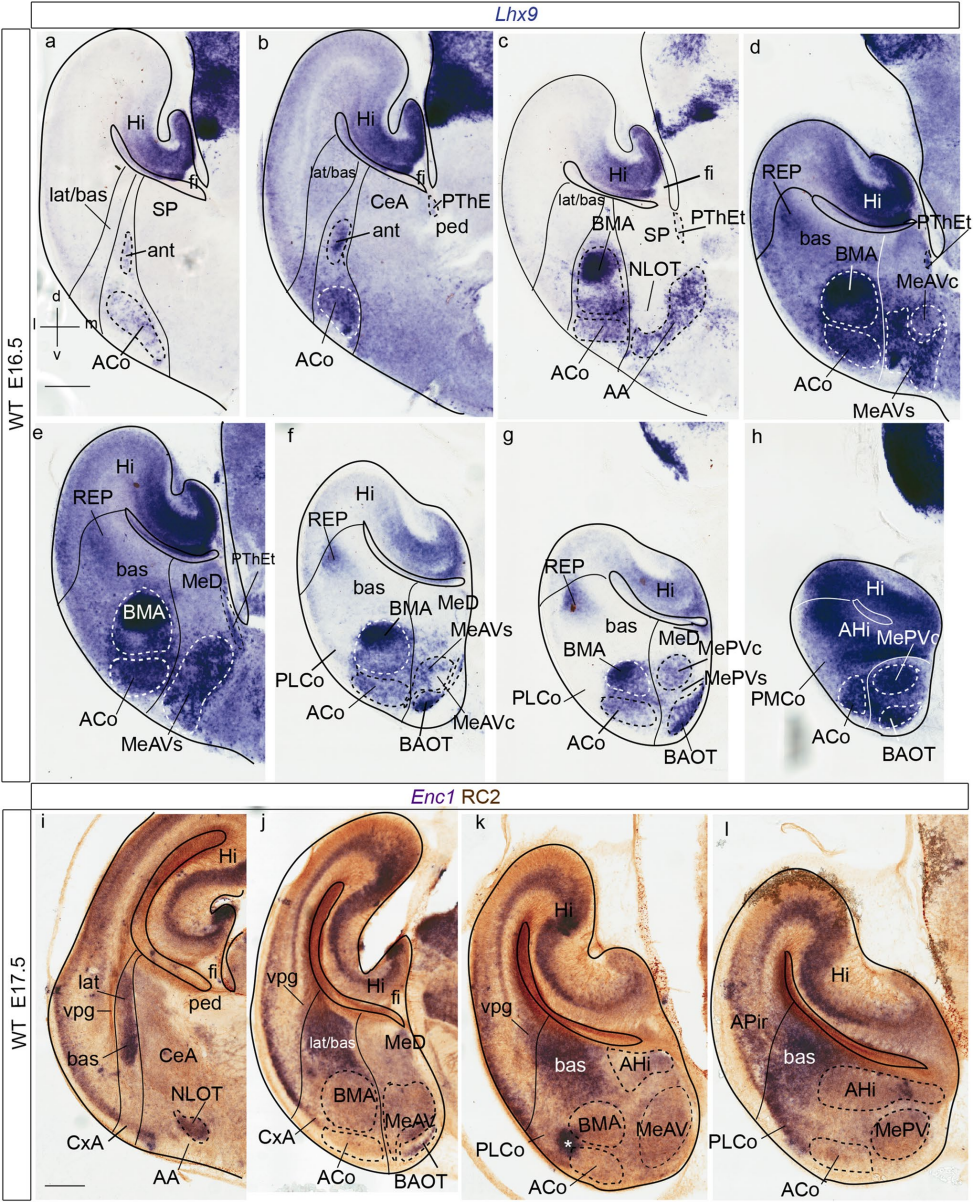
*Lhx9* and *Enc1* expressions in amygdalar radial plane sections in mouse at embryonic stage E16.5, ordered from rostral to caudal; **a** orientation indicated in the bottom left-hand corner. **a**–**h** Expression of *Lhx9* in the amygdalar region. Note in **c** the indentation of *Lhx9*-positive population in AA due to the arrival of the NLOT *Lhx9*-negative migrating stream. **i**–**l** *Enc1* expression counterstained with RC2 (showing radial glia) in mouse amygdalar region. The limits between the *lateral/basal* complex and the cortex, as well as between the *lateral/basal* complex and the *anterior* radial unit, or the latter versus the subpallium are indicated with black or white lines. For abbreviations, see list. Scale bars represent 350 µm (**a**–**l**)

**Fig. 5:**
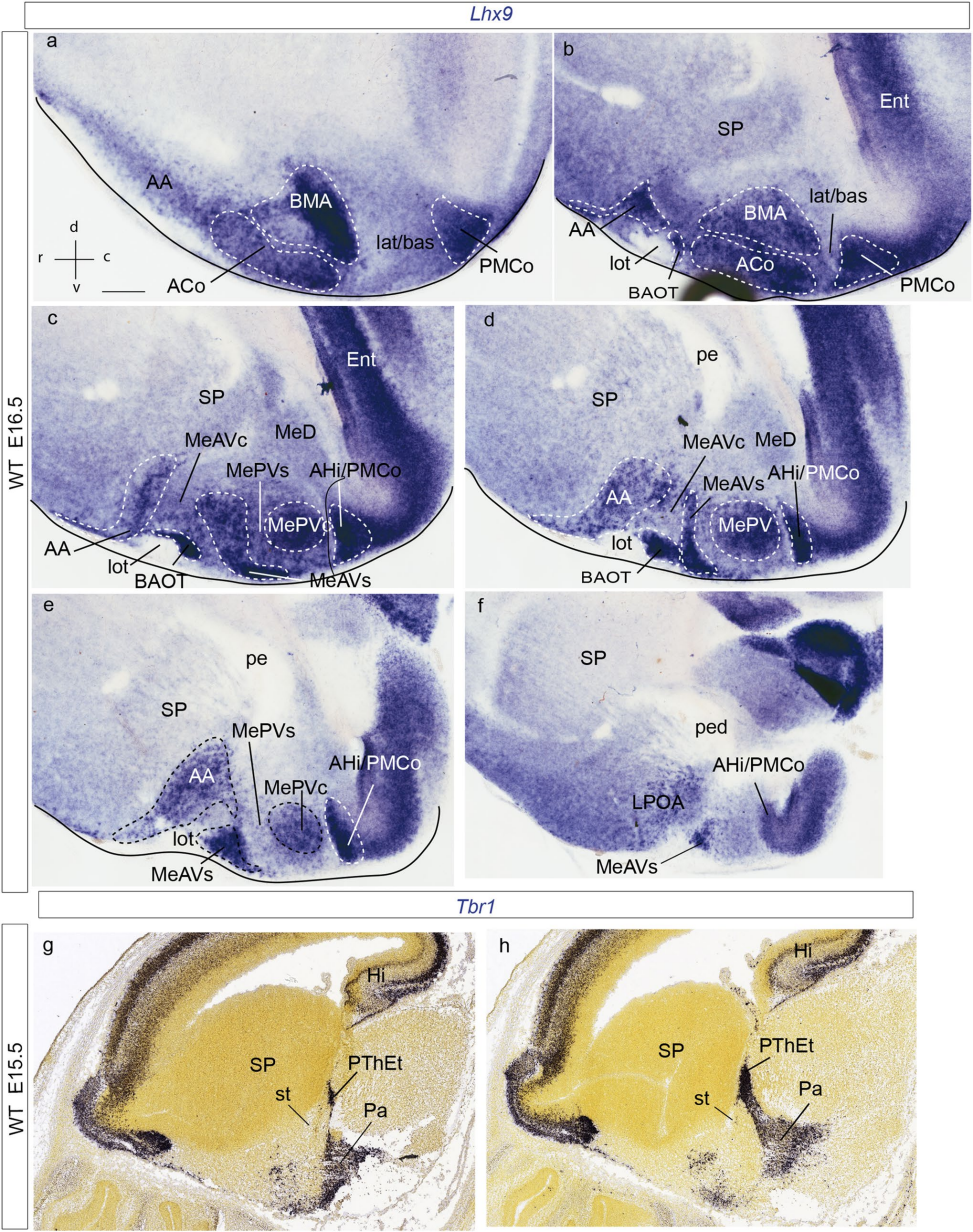
*Lhx9* and *Tbr1* expression in mouse amygdalar region at embryonic stage E16.5 and E15.5 respectively, in sagittal section planes ordered from lateral to medial level; **a** orientation indicated in the down left-hand corner. **a**–**f** *Lhx9* expression at stage E16.5. **g**, **h** Illustration of evaginated (telencephalic) part of prethalamic eminence (PThEt) defined by *Tbr1* expression at stage E15.5 from Website: ©2013 Allen Institute for Brain Science. Allen Developing Mouse Brain Atlas. http://developingmouse.brain-map.org). For abbreviations, see list. Scale bars represent 300 µm (**a**–**h**)

**Fig. 6:**
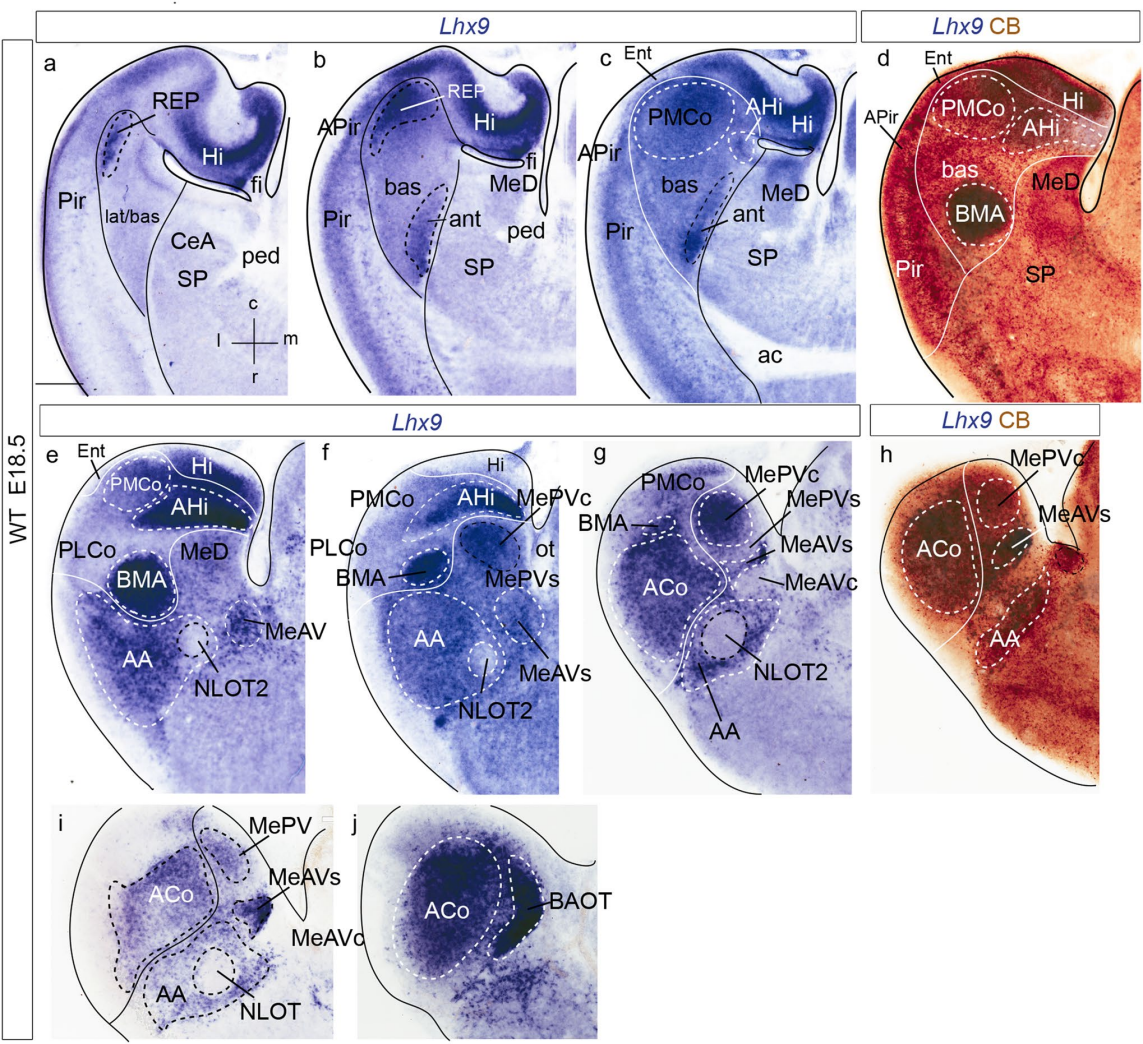
*Lhx9* expression in mouse amygdalar region at embryonic stage E18.5, in horizontal section planes **a**–**h** left side details ordered from dorsal to ventral levels; **i**, **j** mirror-inverted right side details ordered from dorsal to ventral levels, **a** orientation indicated in the bottom right-hand corner; **d**, **h** *Lhx9* expression counterstained with CB. The limits between the *posterior* radial unit and the hippocampus, the *lateral/basal* complex and the cortex, as well as between the *lateral/basal* complex and the *anterior* radial unit, or the latter versus the subpallium are indicated with black lines. For abbreviations, see list. Scale bars represent 300 µm (**a**–**j**)

We next examined amygdalar *Lhx9* expression at intermediate and perinatal stages (E14.5, E16.5 and E18.5) in different section planes (horizontal in Figs. 3 and 6, amygdalar radial in Fig. 4, sagittal in Fig. 5). For comparative purposes, we included *Enc1* expression studied in the amygdalar radial plane at E17.5 (Fig. 4i–l). The text below refers to these three stages analysed together, adding specific stage details when required. The results at perinatal stages were partially advanced in Garcia-Calero et al. (2020).

At intermediate and perinatal stages, the periventricular stratum of the *anterior* radial unit, presumed to be located next to the pallio-subpallial boundary, is *Lhx9*-negative. *Lhx9* expression within the *anterior* unit starts at the deep part of the intermediate stratum with a few scattered *Lhx9* positive cells, and expands importantly at the outer part of the intermediate stratum (site of BMA) and the superficial stratum (ACo) (ant; BMA; ACo; Figs. 3b–g; 4a–g; 5a, b; 6b–j). Coinciding with the reduction of the periventricular and deep intermediate *anterior* radial domain there is a corresponding increase of the periventricular volume of the neighbouring *Enc1*-labelled *lateral* and *basal* radial units (ant; lat/bas; bas; PLCo; Figs. 3b–e; 4a–g, i–l; 5a, b; 6a–f). In addition, numerous *Lhx9*-positive cells spread rostrally from the *anterior* radial unit into the anterior amygdala, more markedly than observed at earlier stages (ant; BMA; AA; Figs. 3c–g; 4c, d; 5a–e; 6e–i).

**Fig. 7:**
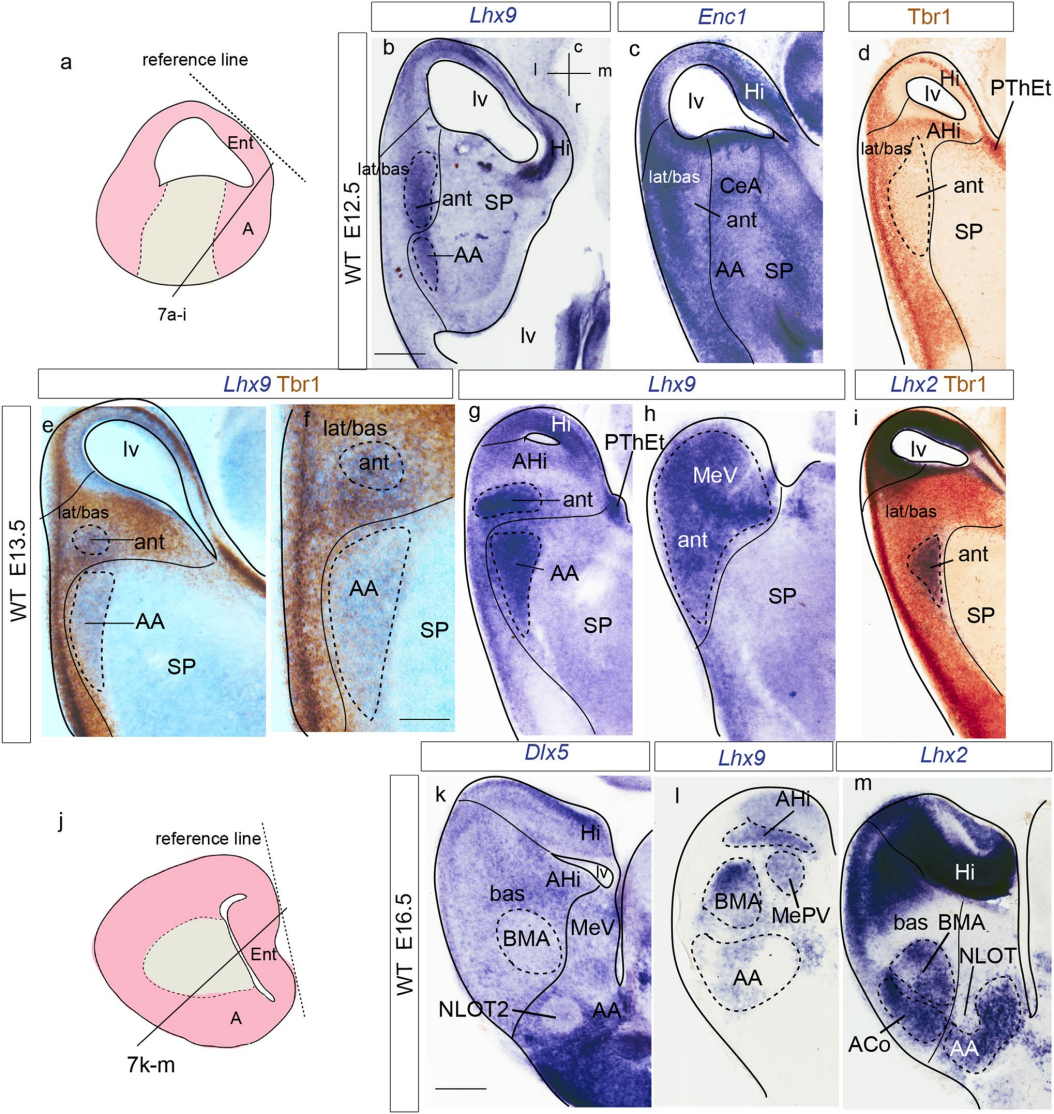
*Lhx9*, *Enc1*, *Lhx2*, Tbr1, *Dlx5* expressions variously compared in mouse telencephalon during development (from E12-5 to E16.5 embryonic stages). **a**, **j** Schematic representation of section planes; **b** orientation indicated in the upper right-hand corner. **b** *Lhx9* expression at *anterior* radial unit (ant) at stage E12.5. **c** *Enc1* expression restricted to lat/bas complex at stage E12.5, laterally to ant. **d** Low Tbr1 protein expression at the ant at stage E12.5. **e**, **f** *Lhx9* expression counterstained with Tbr1 immunoreaction at stage E13.5; note lack of Tbr1 coinciding with the ant radial derivatives (including migrated cells in AA); **f** is a higher magnification detail of the *anterior* radial domain in (**e**); **g**, **h** *Lhx9* expression at E13.5 ordered from dorsal to ventral. **i** *Lhx2* expression restricted to ant radial unit at stage E13.5; section counterstained with Tbr1. **k** *Dlx5*, **l** *Lhx9*, **m** *Lhx2* expressions at stage E16.5. Note in **k** and **m** the advance of the negative NLOT migration stream into the mass of *Dlx5*-positive (subpallial) and *Lhx2*-positive (pallial) AA cells; this section also shows *Lhx2* expression at the BMA and ACo. For abbreviations, see list. Scale bars represent 250 µm (**b**–**e; g**–**i**), 150 µm (**f**) and 300 µm (**k**–**m**)

At the caudal telencephalic pole, the *posterior* radial unit appears also *Lhx9*-labelled, mainly at its periventricular zone, AHi; however, at perinatal stages *Lhx9* transcripts also emerge at the *posterior* intermediate and cortical strata, which form the PMCo primordium; in contrast, the PLCo, the superficial component of the *basomedial lateral* and *medial* subunits, lacks any *Lhx9* signal (AHi; PMCo; PLCo; Figs. 3d–f; 4f–h; 5a–f; 6c–g). At stage E16.5 additional *Lhx9* signal emerges selectively at the periventricular stratum of the *retroendopiriform* radial unit, placed lateral to the negative BLP periventricular nucleus of the *basal* unit (REP; Figs.4d–g; 6a, b). The ventral part of the medial amygdala also shows many positive *Lhx9-*positive cells, in contrast to its negative dorsal part (MeD; MeAV; MePV; Figs. 3c–g; 4d–h; 5c–f; 6b–i). *Lhx9* expression in MeV delineates from E16.5 onwards core and shell regions within the MeAV and MePV nuclei, with a negative core versus positive shell subdivision in MeAV, in contrast to MePV, which displays a *Lhx9*-positive core nucleus and a negative shell (MeAVs; MeAVc; MePVs; MePVc; Figs. 4d-h; 5c–f; 6f–i). There is also a *Lhx9-*positive cell population at the MeD pial surface which we think may be related to the earlier *Lhx9* positive cells dispersing from the PThEt (Figs. 3c-e; 4b–e). This PThEt-related *Lhx9-*positive band extends superficially over superficial MeAV (MeAVs) and may reach the incipient BAOT nucleus (PThEt; MeAVs; BAOT; Figs. 3b–g; 4b–h); this relationship is also visible in sagittal sections (Figs. 5b–f). *Tbr1* expression characterizes the complete PThE domain in the dorsalmost prethalamic area, continuously with apparent tangential migration into superficial MeV and, eventually, BAOT (Figs. 5g, h; Huilgol et al. 2013; Ruiz-Reig et al. 2017; Ruiz-Reig and Studer 2017). At E18.5 the *Lhx9/Tbr1*-positive PThEt patch was not clearly observed. At E16.5, the NLOT migration stream is detectable as a wide *Lhx9*-negative corridor, which starts to indent the caudal limit of the *Lhx9*-positive AA (NLOT; AA; Fig. 4c). At E18.5 the definitive postmigratory *Lhx9* negative NLOT nucleus appears surrounded all around by *Lhx9* positive cells of the AA subpallial region (Fig. 6e–g, i), which also express *Lhx2* (Fig. 7m).

### *Lhx9* amygdalar expression in the molecular context of the developing mouse telencephalon

We compared amygdalar expression of *Lhx9* with pallial mantle Tbr1 protein and subpallial *Dlx5* gene expression in horizontal sections at early and intermediate developmental stages (E12.5, E13.5, E16.5), in order to test its postulated restriction to the pallium (e.g., Tole et al. 2005). Comparison with the presence of *Enc1* and *Lhx2* transcripts at embryonic and perinatal stages was also useful to complete this analysis (Fig. 7).

At stages E12.5/E13.5, the *Lhx9*-positive mantle of the *anterior* radial unit lies within the pallium, even if strictly adjacent to the pallio-subpallial boundary; remarkably, though, this unit displays weaker Tbr1-positive reaction in its mantle layer than other pallial amygdalar regions such as the *lateral, basal* and *posterior* radial units; the latter are selectively identified using the *Enc1* marker, but this does not label the *anterior* radial unit (ant; lat/bas;bas; Fig.7b–g). Similar results were reported previously as regards *Lhx9* and *Tbr1* (Tole et al. 2005; García-López et al. 2008). There is accordingly a qualitative molecular difference between the singular *anterior* radial unit and the other pallial amygdalar units, which remarkably affects among other molecular differences the supposedly fundamental Tbr1 signal (see Tables 1–5 and Suppl. Tables 1 of Garcia-Calero et al. 2020).

Whereas the *anterior* radial unit starts at the ventricle at E12.5, at E13.5 the periventricular and deep intermediate strata of this domain are devoid of positive cells. The latter first appear within the outer part of the intermediate stratum as a small tail-like deep zone medially adjacent to the BLA cell mass, which then expands superficially into the prospective BMA nucleus (ant, BMA; Figs. 7g, h). *Lhx2* expression studied in combination with Tbr1 signal shows an expression pattern similar to that of *Lhx9* at early and late stages, labelling the *anterior* radial unit in overlap with the Tbr1-poor pallial mantle area (Figs. 7i, m), as well as cells in AA which appear to have migrated tangentially from the *anterior* superficial stratum. No signal is found at the NLOT nucleus (Figs. 7m).

At E12.5/E13.5 all the pallial amygdalar radial units are uniformly *Dlx5*-negative areas (data not shown), whereas the AA expresses abundantly *Dlx5* at least up to E16.5 (Fig. 7k). On the other hand, the whole MeA was moderately *Dlx5*-positive up to E14.5 (less than neighboring central amygdala and other striato-pallidal areas; not shown), but appeared to lose this expression at E16.5, possibly as a result of increased numbers of migrated pallial neurons (MeA; Fig. 7k).

Another amygdalar relationship of interest is that with the diencephalic *Lhx9-*positive PThE domain, which shares some pallial markers (e.g., ventricular *Pax6*, and mantle *Tbr1* and *Lhx9*). The latter is a partly evaginated but is essentially an extratelencephalic and hyperdorsal diencephalic area (see Puelles et al. 2020) which contacts the caudal telencephalic pole next to the caudal end of the hippocampus; its dorsal evaginated telencephalic portion (PThEt) finishes attached to the tela at the end of the telencephalic chorioidal fissure (PThE; PThEt; Figs. 7d, g). Some of our data suggest that *Lhx9-*positive eminential cells may invade the superficial stratum of the MeA, possibly reaching the BAOT.

At stage E16.5 we detected some *Dlx5* transcripts in the mantle of the *lateral*, *basal* and *posterior* pallial radial units, apparently corresponding to migrated subpallial cells (Marín and Rubenstein, 2001; AHi; Fig. 7k; results not shown for lat). At the *anterior* radial unit, represented by BMA/ACo, there are less *Dlx5* transcripts than at other amygdalar pallial units (ant; Figs. 7k, l).

## Discussion

**Fig. 8:**
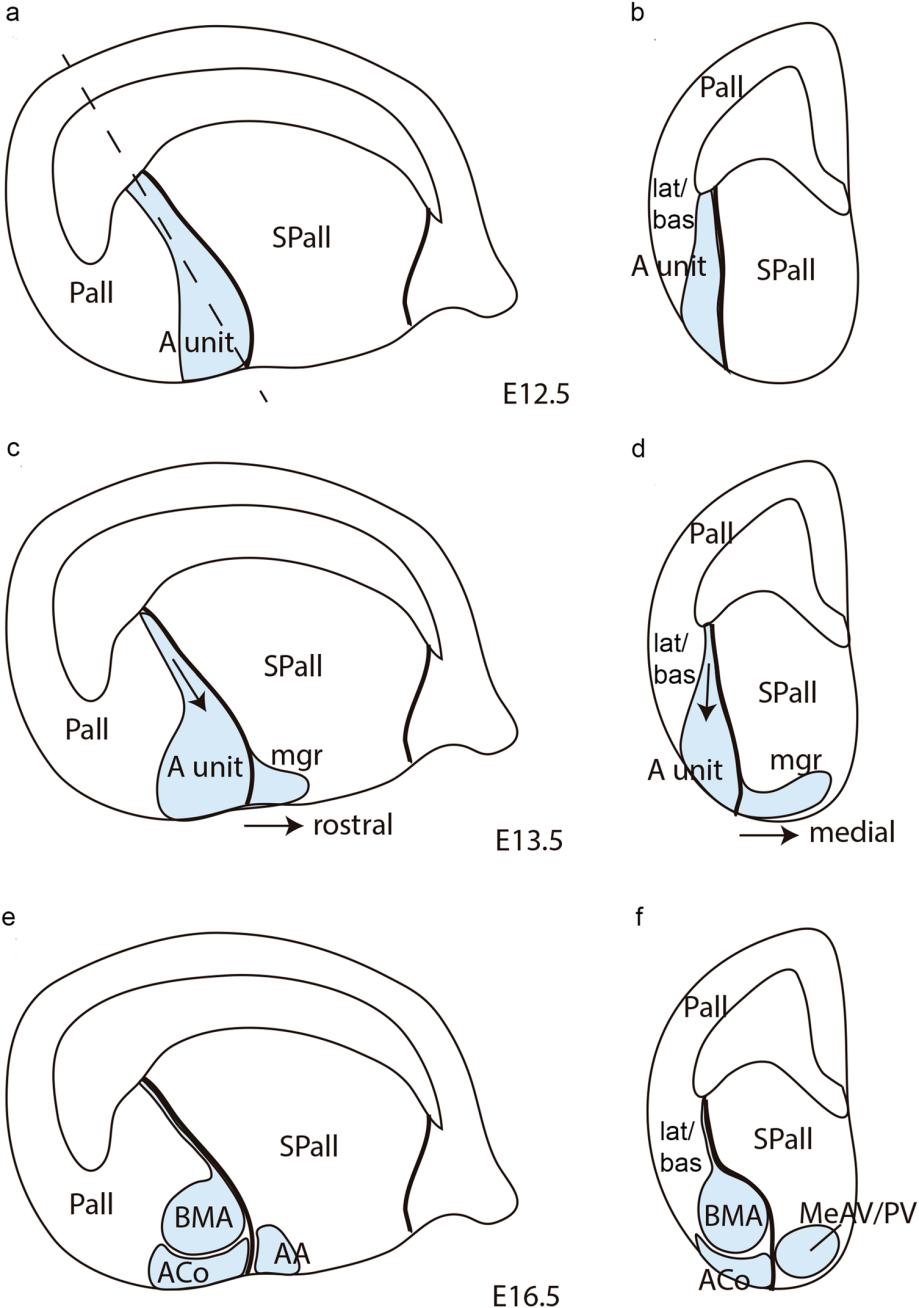
Schema illustrating in sagittal and radial section views the main developmental shape changes shown by the mouse *anterior* amygdalar radial unit (A. unit, light blue), highlighting the latter’s relationship with the telencephalic subpallium and other radial amygdalar domains, such as the* lateral* and* basal* units. **a**, **b** *Anterior* radial unit initial appearance in sagittal and radial amygdalar section planes in mouse embryos at stage E12.5. The *anterior* radial unit appears as a compact structure extending radially from the ventricle to the pial surface, close to the pallial/subpallial boundary. The *lateral* and *basal* radial units are located lateral to the *anterior* radial unit in (**b**). The dash line in **a** shows the radial amygdalar section plane used in **b**, **d**, **f**. **c**, **d** *Anterior* radial unit shape and postulated intrinsic radial cell migration movements in mouse embryo sagittal and radial amygdalar sections at stage E13.5. The *anterior* radial unit shape shows a narrowing in the periventricular stratum, presumably due to radial cell migration from this region to the intermediate and superficial strata (black arrow). There is also an apparent tangential cell migration of identically labeled cells spreading from the unit’s superficial stratum into rostrally and medially adjacent amygdalar subpallium (mgr). **e**, **f** Definitive shape of the *anterior* radial unit in sagittal and radial amygdalar sections of mouse embryos at stage E16.5, after full depopulation of its periventricular stratum (where only a thin radial glial palisade remains) and definition of its derivatives, the intermediate basomedial nucleus (BMA) and the superficial anterior cortical nucleus (ACo). The derivatives of the previous tangential migrations into the anterior and medial amygdalar subpallium are also represented (anterior amygdalar nucleus, or AA, and anteroventral and posteroventral medial amygdalar nuclei, or MeAV/MePV)

Our main goal in this work was a detailed description of *Lhx9* expression in the amygdalar area as a means to explore its molecular partitions, and in particular the derivatives of the *anterior* radial unit. We consider this gene a partially selective marker of the *anterior* radial unit (since it labels also other amygdalar structures) on the basis of our recent genoarchitectural analysis of the pallial amygdala (Garcia-Calero et al. 2020; summary of radial units in Table 1). The *anterior* radial unit is singular in that it lacks a populated periventricular stratum due to superficial translocation of all its derivatives. We followed the progress of this phenomenon during telencephalic development, and noted changing relationships with surrounding pallial structures, while retaining the primary contact with the subpallium (Fig. 8). We also illustrated the development of other amygdalar regions found labelled by *Lhx9* signal outside the *anterior* radial unit, such as the *posterior* radial unit (AHi/PMCo), the periventricular retroendopiriform nucleus (REP) lying lateral to the BLP nucleus, the bed nucleus of the accessory olfactory tract (BAOT), and parts of the anterior and medial subpallial amygdala. We will discuss below the relationships of *Lhx9* transcripts with other molecular markers potentially distinguishing a ventral pallium-like sector in the pallial amygdala and also consider in the context of our recent amygdalar radial model the conventional notion of a migratory cortical origin of diverse amygdalar pallial sectors (Medina et al. 2004; Deussing and Wurst 2007). Our conclusion is that it is not meaningful to extrapolate subdivisions of the cortical pallium by merely *assumed* migrations to the topologically separate amygdalar pallium, irrespective of shared gene markers. Both the cortex and the amygdala have distinct patterns of intrinsic subdivision with differential molecular profiles.

### *Lhx9* labels the *anterior* radial domain in the mouse pallial amygdala

*Lhx9* gene belongs to the LIM-homeodomain gene family, a group of transcription factors which play important roles during embryonic development in vertebrates, including CNS formation (Tuschida et al. 1994; Retoux et al. 1999; Bertuzzi et al. 1999; Nakagawa and O’Leary 2001; Bachy et al. 2001; Shirasaki and Pfaff 2002; Bulchand et al. 2003; Remedios et al. 2004; Tole et al. 2005; García-López et al. 2008; Abellán et al. 2009, 2010, 2013, 2014; Desfilis et al. 2018). Combinatorial expression of some LIM genes defines cell, nuclear and regional identities during development of spinal cord, thalamus and telencephalon (Nakawaga and O´Leary 2001; Shirakassi and Pfaff 2002; Remedios et al. 2004; Tole et al. 2005; García-López et al. 2008; Abellán et al. 2009, 2010, 2013, 2014).

A role has been proposed for LIM-genes also in mouse amygdalar parcellation, mainly as regards its pallio-subpallial subdivision: *Lhx9* and *Lhx2* transcripts are described as characteristic of pallial amygdala, whereas *Lhx6* is expressed in the subpallial amygdala at embryonic stages (Remedios et al. 2004; Tole et al. 2005; Choi et al. 2005; García-López et al. 2008; Abellán et al. 2009; 2013; Medina et al. 2017). Moreover, the whole cortical ventral pallium (i.e., the olfactory pallial sector; Puelles, 2014, 2017; Puelles et al. 2019) and a hypothetic ventropallial amygdalar subregion have been co-defined by selective *Lhx9* expression (Tole et al. 2005; García-López et al. 2008; Abellán et al. 2009; Medina et al. 2017). Our present results lead us to discrepate with these two conclusions. First, we find both *Lhx9* and *Lhx2* signals not only in the pallial amygdala, but also, independently, in parts of the subpallial amygdala (AA, MeA). Secondly, our data suggest that *Lhx9* does not label initially the entire cortical ventral pallium, nor all possible ‘ventropallial’ parts of the amygdala according to the Puelles et al. (2016a) analysis. Indeed, its amygdalar signal is always restricted over time, first to the *anterior* unit primordium at early stages, and then to the mature derivatives of the *anterior* radial amygdalar unit, some of which apparently invade tangentially the AA and MeA subpallial regions.

*Lhx9* labels several nuclei in the amygdalar domain, namely BMA/ACo in the *anterior* radial unit, AHi/PMCo in the *posterior* radial unit; the periventricular REP part of the *retroendopiriform* radial unit, and various possibly tangentially migrated cell groups in the subpallial AA and MeA (Remedios et al. 2004; García-López et al. 2008; Abellán et al. 2009, 2013; Garcia-Calero et al. 2020). Our developmental study corroborates previous reports on the existence of an early domain with full radial *Lhx9* expression, found next to the pallio-subpallial boundary (e.g., Tole et al. 2005; García-López et al. 2008; Fig. 8). However, we think that this labelling represents only the *anterior* radial unit of Garcia-Calero et al. (2020), together with some tangentially migrated cells, rather than the cortical ventral pallium in general. That is, the *Lhx9* signal shown by these authors is always restricted to a particular radial unit of the *amygdalar* pallium, and is not significantly present in *cortical* ventral pallium.

Interestingly, Tole et al. (2005) reported that the *Lhx9*-positive radial cell stream they identified adjacent to the pallio-subpallial boundary overlaps with a mantle domain showing low *Tbr1* expression at early mouse embryonic stages; sagittal sections of the *Tbr1* E13.5 specimen illustrated at the Allen Developing Mouse Brain Atlas clearly show that this low *Tbr1-*signal area is restricted to amygdalar pallium. Guided by its position bordering the subpallium, and possibly following notions developed by Medina et al. (2004) in search of amygdalar correspondences, these authors interpreted that this peculiar *Lhx9-*positive and low *Tbr1* band represented the ventropallial *migratory stream*, that is, the postulated active movement of ventropallial cells born at the cortical ventral pallium sector of the hemisphere (VPall as defined by Puelles et al. 2000 and Medina et al. 2004) into the amygdala. However, the cortical VPall shows at all early embryonic stages strong *Tbr1* expression. For similar reasons, the *Lhx9*-negative mantle region found lateral to the amygdalar *Lhx9* domain, which shows discrete *Emx1* and *Cdh8* expression, was wrongly ascribed to the lateral pallium (LPall), given that recent additional research with the claustro-insular markers *Nr4a2* and *Cyp26b* indicated that the updated claustro-insular LPall does not extend into the amygdalar field (Puelles 2014, 2017). This implies that this lateral amygdalar pallial locus must be a separate and molecularly distinct portion of the pallial amygdala, presently estimated to include the *lateral* and *basal* radial units.

Following similar early and imperfect criteria, other authors proposed that early ventropallial expression of *Lhx9* extended to *caudal* ventral pallium levels encompassing the whole, or a large part, of the pallial amygdala domain (García-López et al. 2008; Abellán et al. 2009; Medina et al. 2017). Since it was becoming evident by then that cortical sectors of VPall did not express *Lhx9* at middle and late embryonic stages, it was implicitly assumed by those interested in this topic that *Lhx9* possibly was downregulated during development in the whole rostral VPall, as well as in the basolateral amygdalar nuclear complexes (also thought to belong to VPall at least in part; Puelles et al. 2016a), while only some nuclear entities such as BMA and ACo retained *Lhx9* expression (García-López et al. 2008; Abellán et al. 2009; Medina et al. 2017).

We also observed that full radial *Lhx9* transcripts typically appear next to the pallio-subpallial boundary at E12.5, in a region which is poor in Tbr1 protein (as first shown by Tole et al. 2005; Fig.8a, b). However, our morphologic interpretation differs, because we consider the data under the novel light of the radial model of the pallial amygdala (Garcia-Calero et al. 2020; Table 1). This postulates a distinct *anterior* radial unit which is primarily independent from other amygdalar pallial partitions, both in terms of glial architecture and molecular profile, and lies intercalated between the *lateral*/*basal* amygdalar radial units and the local subpallium. As shown by present results, this radial region accumulates its cells via radial migration restricted to the pallial amygdala in its outer-intermediate and superficial layers as of E13.5 (Figs. 8c, d), producing the BMA and ACo, respectively (Figs. 8e, f). The corresponding periventricular domain remains represented only by a glial palisade, an aspect hardly visualized in Nissl-stained material or in ISH genoarchitectural material (see corresponding experimental DiI labeling of radial glia in Garcia-Calero et al. 2020). We compared from E12.5 onwards the amygdalar *Lhx9-*positive *anterior* radial unit with selective *Enc1* labelling of the adjacent *lateral*/*basal* radial unit complex, discovering that the *lateral* and *basal* amygdalar radial unit primordia already exist next to the amygdalar *Lhx9*-positive domain, which coincides with low Tbr1 signal at E12.5, and can be recognized steadily at the same site subsequently. Eventually the *lateral* and *basal* radial units develop massive periventricular nuclei (L, BLP and BMPM/BMPL), jointly with intermediate populations (LI, BLA, BLI, BMIM, BMIL) and superficial aggregates (CxAR, CxAC, PLCo) in contrast to the superficialized *anterior* unit (BMA, ACo; data from Garcia-Calero et al. 2020). Such relationships of *Lhx9*+*/low Tbr1* and *Enc1*+ labelled fields only obtain at amygdalar levels, in contrast with the cortical pallium. These results imply that the full radial early *Lhx9-*positive domain observed at E12.5 represents only the prospective *anterior* radial unit, and not all potentially ventropallial analogs of the pallial amygdala, which include at least part of the *lateral/basal* complex according to *Dbx1*-related findings (Medina et al. 2004, 2017; Puelles et al. 2016a). Moreover, we conclude that probably no part of (cortical) ventral pallium ever expresses *Lhx9*, given that this domain strongly co-expresses *Tbr1* throughout development (Puelles et al. 2000).

We believe that the interpretive error incurred by the authors cited above in assuming that the early amygdalar *Lhx9*-positive *anterior* unit domain represented partly the preamygdalar (cortical) VPall sector was possibly due to the fact that at early developmental stages the amygdalar region occupies a larger proportional longitudinal extent of the hemisphere than at later stages, as is suggested by comparison of our early and late sagittal sections (see our Figs.1j, k and 5a–e); unfortunately, sagittal sections apparently were not examined by either Tole et al. (2005) or García-López et al. (2008). This morphogenetic change is probably due at least in part to differential growth of the ganglionic eminences and the cortex relative to the amygdalar complex.

It may be significant to note that the *lateral/basal* amygdalar complex does *not* contact the pallio-subpallial boundary, due to the physical intercalation of the *anterior* and *posterior* radial amygdalar units between the subpallium and the remaining amygdalar pallium (Garcia-Calero et al. 2020; remarkably, these two radial domains express separately *Lhx9*). A para-striatal topology is supposed to be a uniform characteristic of cortical VPall. The *lateral/basal* amygdalar complex (containing four full radial subunits; Table 1) would then represent topologically an unique *Lhx9*-negative and *Enc1*-positive region existing within amygdalar pallium, for which there is no apparent counterpart outside the amygdala (note a LPall component was excluded by Puelles, 2014, 2017).

Another Lim-homeodomain transcription factor, *Lhx2,* shows a quite similar expression pattern than its paralog *Lhx9* at the *anterior* radial unit, though it was suggested to expand more laterally than *Lhx9* (e.g., Fig. 1 in Tole et al. 2005). In our hands, strong *Lhx2* signal observed next to the amygdalar subpallium was strictly restricted to the *anterior* radial unit nuclei or to *anterior* cells suspected to have migrated tangentially into amygdalar subpallium (Figs.7m, 8d–f; Garcia-Calero et al. 2020). This transcription factor may have redundant functions at this locus with respect to *Lhx9* (Remedios et al. 2004). On the other hand, loss of function of *Lhx2* reportedly produces lack of migration of the NLOT2 nucleus (Remedios et al. 2007; see also Garcia-Calero et al. 2021, where we deal specifically with this migration).

### *Lhx9* expression at the amygdalar caudal pole

Amygdalar *Lhx9* transcripts also appear at caudal telencephalic levels, where they encompass a large part of the caudal telencephalic pole. Apart of standard intermediate/superficial elements derived from the *anterior* radial unit (ACo and BMA), the marker also appears at the *posterior* unit (particularly AHi of the AHi/PMCo complex), as well as in parts of the neighbouring hippocampal complex (medial pallium) and, separately, at the periventricular component of the *retroendopiriform* radial unit (Garcia-Calero et al. 2020) and the BAOT superficial nucleus. None of these extra expression domains seems close enough topographically to be considered an extension of the *anterior* radial unit.

On the other hand, there is visible labelling continuity between the ACo (superficial *anterior* pallial element) and the rostrally adjacent anterior amygdala (AA), which is widely held to be intrinsically subpallial in nature, as is corroborated by our *Dlx5* mapping data. The *Lhx9*-positive AA cell population is accordingly presumably glutamatergic, secondarily mixed in with the local subpallial GABAergic neurons. The AA finally surrounds the separately migrated *Lhx9/Lhx2*-negative NLOT (Remedios et al. 2007). This apparent invasion of AA from the *anterior* pallial unit is already incipient at E12.5, and increases thereafter up to E16.5 (Figs. 8c, e).

Secondly, the *Lhx9/Lhx2*-positive ACo/AA cell population also shows marked continuity with similar cells found at the MeA area of the subpallial amygdala, incipient already at E12.5 and E13.5 (Figs. 1, 2; 8d, f). As development advances, the initially single positive MeA population separates into labelled cells at the MeAV and MePV nuclei (Fig. 3d–g; 4e–h; 5d, e; 6e–i; 7l). Whereas the MeAV shows cells with both *Lhx9* and *Lhx2* transcripts, the MePV does not contain *Lhx2*-positive cells (Fig. 7m). Once stabilized at the MeAV, *Lhx9* cells predominantly surround its weakly labelled core subdivision, forming a positive shell around it. In contrast, the opposite pattern characterizes the MePV, where essentially a *Lhx9*-positive core and a weakly labelled shell are displayed.

We already conjectured before that this medial amygdala pattern probably needs to be interpreted as part of a tangential migration out of the *anterior* radial amygdalar unit into amygdalar subpallium (Garcia-Calero et al. 2020). Bupesh et al. (2011) previously postulated likewise a *Lhx9*-positive ventropallial migration into MeAV; however, we restrict its origin to the *anterior* amygdalar unit, without implicating an origin at the cortical VPall. The MePV nucleus must be distinctly heterogeneous in its cellular composition, since it reportedly is also tangentially invaded by *Otp*-positive cells from the paraventricular hypothalamic area (García-Moreno et al. 2010) and by *Shh*-positive cells from the septocommissural area (Hirata et al. 2009; Carney et al. 2010; Bupesh et al. 2011; Lischinsky et al. 2017).

A parallel observation was the development from intermediate embryonic stages onwards of the half-moon-shaped small superficial BAOT nucleus, which also expresses strongly the *Lhx9* marker, and is found at the rostromedial border of the MeAV (Fig. 4f–h; 5c–e; 6j). However, lack of *Lhx2* at the BAOT, and other differential molecular data (Table 4 and Suppl. Table 1 in Garcia-Calero et al. 2020) do not support an origin of this nucleus also at the amygdalar *anterior* radial unit. The nearest alternative *Lhx9*-positive forebrain regions are the *posterior* amygdalar radial unit and the evaginated or ‘telencephalic’ part of the prethalamic eminence (marked PThEt in our Figures). Resolving this issue will require a specific investigation.

Ruiz-Reig et al. (2018) observed that the caudal telencephalic pole was negative for subpallial markers such as *Gsx2* and *Ascl1*, but displayed transcripts of the pallial genes *Pax6* and Tbr2 protein at stage E12.5. This agrees with the conventional idea that the subpallial caudal ganglionic eminence (CGE) ends short of the caudal hemispheric pole proper, so that pallial tissue surrounds caudally the CGE (review in Puelles et al., 2013, 2016b). The pallial nature of the remnants of the *basal*, *anterior* and *posterior* radial units still present at the caudal telencephalic pole is well accepted in the literature (from Swanson and Petrovich 1998 to Garcia-Calero et al. 2020). In addition, Ruiz-Reig et al. (2018) presented evidence suggesting that an *Ebf3*+/Tbr1+ cell population migrates from an unidentified amygdalar pallial origin into to MePV (probably forming what we identified as MePVs). These authors also postulated an extra ‘caudo-ventral’ part within the amygdalar pallium which contributes cells to the MeAV (Ruiz-Reig et al. 2018); this should not be confused with the ‘ventro-caudal’ or ‘ventrolateral caudal’ amygdalar pallial part of Medina et al. (2017), nor with our *anterior* radial unit.

We now regard earlier tentative pallial partitions based on standard coronal sections (oblique to amygdalar radial glial structure) as insufficiently documented with regard to their molecular borders and derivatives, particularly as regards the amygdala. Significantly, the postulated migratory streams supposedly leading from various cortical pallial sectors into the amygdala (Medina et al. 2004; Tole et al. 2005; Remedios et al. 2004, 2007; Puelles et al. 2016a) have not received experimental corroboration. The amygdalar radial model (Garcia-Calero et al. 2020) offers a solid alternative schema of intrinsic amygdalar radial development for resolving this problem. In conclusion, most of the neural structures identified at the caudal telencephalic pole show a pallial molecular profile, eventually including *Lhx9* expression (either via autochthonous differentiation within *anterior, posterior* or *rep* amygdalar pallium, or via tangential migration of pallium-originated cells into MeA).

### Variety of molecular subdivisions in the amygdalar pallial field

Molecular pallial regionalization accompanied molecular distinction of pallium and subpallium domains in the telencephalon (Smith-Fernández et al. 1998; Puelles et al. 2000). Classic anatomic studies were based upon a tripartite pallium model (medial hippocampal pallium, dorsal neopallium, and lateral olfactory pallium; reviewed in Striedter 1997). This model clearly demanded correction, once *two new molecular pallial sectors* respectively positive and negative for *Emx1* signal were discovered inside the old olfactory pallium (Smith-Fernández et al. 1998; Puelles et al. 2000); this led to the first tetrapartite pallium model (MPall, DPall, LPall, VPall, where the old ‘lateral’ pallium =  was equated to the new LPall + *Emx1*-negative VPall; Puelles et al. 2000). It was assumed at the time that cortical and amygdalar pallium was unitary, implying that the new LPall and VPall sectors should extend into the amygdalar complex, forming a classic ‘claustroamygdalar’ continuum (e.g., Holmgren 1925; Kuhlenbeck 1973). Drawing on this assumption Medina et al. (2004) and Tole et al. (2005) explored in detail the amygdalar region, using a variety of subpallial and pallial markers (see also Martínez-García et al. 2008, 2012). Among a good number of solid findings, particularly on *Dbx1*, *Ngn2*, *semaphorin5A*, *Emx2* expression at the cortical and amygdalar VPall, other results, notably on LPall, require in retrospect reinterpretation, given it was later discovered that neither the claustrum nor selective claustro-insular molecular markers extend into the amygdala (Puelles 2014, 2017; Puelles et al. 2019). Mapped markers whose amygdalar expression needs to be re-evaluated include *Emx1*, *Lhx9*, and *Cdh8*.

The *Dbx1* gene was first reported as a ventropallial marker due to its labelling of a longitudinal ventricular microzone found next to the pallio-subpallial boundary throughout the hemisphere (Yun et al. 2001; Medina et al. 2004; Bielle et al. 2005). As determined by progeny analysis (see below) the mantle of this domain corresponds to the *Emx1*-poor, *Sfrp2*-positive, *Ngn2*-positive, *semaphorin5A*-positive and *Emx2*-positive VPall sector (Smith-Fernandez et al. 1998; Puelles et al. 2000; Kim et al. 2001; Gorski et al. 2002; Medina et al. 2004; Tole et al. 2005). Its cortical pallial derivatives were subsequently determined to include the olfactory cortex, the ventral endopiriform nucleus, the bed nucleus of the external capsule (Medina et al. 2004; Bielle et al. 2005; Hirata et al. 2009; Waclaw et al. 2010; Puelles et al. 2016a).

An analysis of *Dbx1*-related progeny studied by Puelles et al. (2016a) in *Db*x*1*-LacZ transgenic mice gave the impression that amygdalar *Dbx1*-derivatives predominate in the BMA, CxA and ACo nuclei, as well as in *anterior parts* of the L and BLA nuclei. Such derivatives were relatively scarce in the corresponding *posterior parts* of the amygdala (i.e., caudal L and BLA, BLP and BMP, as well as PLCo, AHi and PMCo). In fact, what was shown at P60 was a caudally decreasing proportion of LacZ-positive neurons mixed with negative neurons at all section levels. In addition, the posterior amygdalar areas are rich in *Emx1* transcripts and *Emx1*-cell linage derivatives, a gene marker poorly expressed in cortical VPall (Puelles et al. 2000; Gorski et al. 2002; Medina et al. 2004; Remedios et al. 2007; Cocas et al. 2011). Though distinct *Emx1* signal was found at the BLA nucleus, leading to its initial ascription to lateral pallium (Medina et al. 2004), recently the lateral pallium was radically re-defined as the radial territory which encompasses selectively the molecularly distinct claustro-insular formation, which does not extend into the amygdalar area (Puelles 2014, 2017; Puelles et al. 2019). Taken together, these results reveal molecular similarity between parts of the pallial amygdala with three cortical pallial sectors. Namely, the rostral ant/lat/bas amygdalar subdivisions showing a higher proportion of *Dbx1*-LacZ-positive cells are comparable molecularly to the *ventral* pallium. In contrast, the *Emx1*-positive posterior (periventricular) and basolateral amygdalar parts showing a low proportion of *Dbx1*-LacZ-negative cells might be tentatively ascribed to a novel *ventrolateral caudal* or *ventrocaudal* amygdalar pallium sector (Puelles et al. 2016a). Finally, the *posterior* AHi/PMCo complex would be largely devoid of *Dbx1* signal and might be compared on molecular grounds with the *medial* pallium (Abellán et al. 2014; Puelles et al. 2016a; Medina et al. 2017; Desfilis et al. 2018).

Ruiz-Reig et al. (2018) identified an exclusively amygdalar *caudoventral* subdivision characterized by *Gdf10*, *Sfrp2* and *Fgf15* expression, which they think is continuous but not identical with the amygdalar ventral pallium, and has no clear resemblance with any of the cortical pallial sectors. It was claimed by Ruiz-Reig et al. (2018) that their *caudoventral* amygdalar subdivision, whose ventricular zone does not express *Dbx1,* and contributes cells to a subregion of the MePV, does not correspond to the *ventrocaudal* pallium proposed in chicken, lizard, and mice by Abellán et al. (2014) and Medina et al. (2017).

Irrespective of the foregoing summary of available molecular data on pallial amygdala, any division system of the pallial amygdala which does not take into consideration its primary radial histogenetic organization, related to its intrinsic (rather than extrinsic cortical) ventricular progenitor partitions must be reexamined. Notably, all the notions of molecular amygdalar subdivision cited above were deduced from *coronal sections oblique to radial amygdalar organization* as demonstrated experimentally by Garcia-Calero et al. (2020). Indeed, previous anatomic work on amygdalar structure ranging back to Johnston (1923) was generally oblivious of where lies the amygdalar ventricular zone where distinct cell populations are produced over time (Garcia-Calero et al. 2020). This criticism applies also to work from our own laboratory (e.g., Puelles et al., 2000, 2016a; Medina et al. 2004; García-López et al. 2008). The so-called ‘posterior’ and ‘anterior’ amygdalar pallial parts discussed by Puelles et al. (2016a) consist of artefactually separated *periventricular* versus *superficial* parts of various radial amygdalar units (Garcia-Calero et al. 2020). This traditional but conceptually wrong approach introduces a gross error in the assumptions used regarding the position of the ventricular zone that generates the different amygdalar cell groups studied (distorting among other concepts the meaning of the descriptors ‘rostral/anterior’ and ‘caudal/posterior). Molecular profiles need to be examined in proper histogenetic context, which eventually implies stratified radial structure (Nieuwenhuys and Puelles 2016; Garcia-Calero and Puelles 2020). It does not make sense to continue using standard coronal sections to subdivide the pallial amygdala molecularly, and older work doing so needs to be reinterpreted.

We accordingly have reinterpreted the Puelles et al. (2016a) data consistently with the new amygdalar radial model (Garcia-Calero et al. 2020). The first step would be to correlate early amygdalar expression of *Dbx1* at E10.5-E11.5 with observations at later stages. Mantle derivatives of the *Dbx1-*labelled microzone can be studied in a second step. Work by Medina et al. (2004), Bielle et al. (2005), Teissier et al. 2010 and Puelles et al. (2016a) (e.g., see their Fig. 1a–c) showed that the early weakly *Dbx1*-positive *cortical* ventricular stripe found adjacent to the subpallial striatal territory (representing the cortical VPall) extends into amygdalar territory and finally bends medialwards around the end of the caudal ganglionic eminence (CGE), finally contacting the dorsal extension of the hypothalamic paraventricular area (Pa); the latter is represented early on by a longitudinal alar hypothalamic band of *Dbx1* signal (see the Allen Developing Mouse Brain Atlas E11.5 data for *Dbx1*). The dorsal Pa displays at its caudal end, next to the bordering prethalamic eminence, a spike-like dorsal expansion which penetrates the hemispheric stalk through the floor of the interventricular foramen, then follows the floor of the terminal sulcus, until it meets the amygdalar *Dbx1*-labelled band at its caudal end (this hypothalamic spike reaching the evaginated amygdalar region was observed already at E9.5 by Fan et al. 1996, who used *Sim1* mapping of the paraventricular area; it was illustrated schematically in Fig. 3 of Puelles and Rubenstein, 2003, and Fig. 10 of Puelles and Rubenstein 2015; recently we proposed to call it the *hypothalamo-amygdalar corridor or HyA*; Garcia-Calero et al. 2021). Note *Dbx1-*derived progeny clearly appears periventricularly at the floor of the terminal sulcus in E14.5 embryos, as shown by Puelles et al. (2016a) in their Figs. 2a–c. A similar para-subpallial distribution of an amygdalar stripe expressing *Sfrp2* was previously found to contour caudally the CGE and reach the alar hypothalamus via the bottom of the terminal sulcus, that is, the HyA (Kim et al. 2001). These data jointly indicate that there exists an early molecularly distinct neuroepithelial band that accompanies the pallio-subpallial boundary along both the cortical and amygdalar parts of the pallium, and also connects finally with the dorsal part of the alar hypothalamus, which limits with the overlying telencephalic subpallium (first lateral to the LGE, and then around or under CGE and MGE). Given this band of tissue primarily positive for both* Dbx1* and* Sfrp2*, there is no need for a cortical migration to produce corresponding amygdalar cell populations. One expects no change in this early topology, irrespective of morphogenetic deformations due to advancing telencephalic growth.

The next step is to approach the subsequent fate (derivatives/progeny) of the amygdalar part of the *Dbx1*-positive band. Given the existence of five pallial amygdala radial units (*ant/lat/bas/post/rep*; see Table 1; Garcia-Calero et al. 2020), it would be possible in principle that the amygdalar part of the *Dbx1/Sfrp2*-positive band corresponds solely to the *anterior* and *posterior* radial units, which are the ones contacting directly the subpallium; this would leave the *lateral, basal* and *rep* amygdalar units outside the topological para-subpallial position. Following this notion, one would expect accordingly *Dbx1*-progeny to be *restricted* to the BMA/ACo (*ant* derivatives) and AHi/PMCo (*post* derivatives). However, this possibility is apparently negated by the *Dbx1*-LacZ progeny data of both Waclaw et al. (2010) and Puelles et al. (2016a).

The latter authors indeed reported* Dbx1-*labelled *anterior* unit progeny (BMA/ACo), but also abundant labelled progeny at some parts of the *lateral* and *basal* radial units (L, CxA and the anterior part of BLA). Remarkably, they did not recognize *Dbx1* progeny at nuclei derived from the *posterior* unit (AHi/PMCo). However, with more experience of amygdalar structure than we had then (due to our work in Garcia-Calero et al. 2020, 2021, Garcia-Calero and Puelles 2020, and present report), we would reinterpret the labelled cell mass which Puelles et al. (2016a) tagged as ‘MePV’ in their Fig. 2d (E14.5) as corresponding to the AHi/PMCo complex, found caudal to the MePV proper. Their Fig. 5b (E18.5), illustrating a section caudal to the MeA seen in Fig. 5a that shows ventricular labelling, may be reinterpreted similarly as corresponding to the AHi/PMCo *posterior* complex). At E18.5, Puelles et al (2016a) did not observe labelling of PLCo, the superficial subunit of the *basomediolateral/basomediomedial* radial *bas* subunits (see present Table 1), but the corresponding periventricular formation appears strongly labelled (tagged BM) in their Fig. 4d, e, and radial streams of labelled cells spread out from the periventricular stratum into a superficial locus tagged ‘CxA’ (same Figures). We think that this actually corresponds to the missing PLCo. The periventricular BLP nucleus of the *basolateral* radial *bas* subunit was described as unlabelled, but its periventricular mass at E18.5 was also labelled distinctly, misidentified as ‘L’ (Puelles et al. 2016a; their Fig. 4d, e). Interestingly, ventricular labelling at the caudal BLP locus is continuous with the extra-amygdalar ventricular zone of the cortical olfactory VPall (tagged in the cited Figs. as ‘VPne’), thus necessarily including also the intercalated *rep* domain (Garcia-Calero et al. 2020).

Such reappraisal of the *Dbx1* progeny material (which is also roughly consistent with data from Waclaw et al. 2010), plus the observation that the postnatal pallial amygdala shows *Dbx1*-labelled cells in *all its nuclear derivatives* (mixed in a varying proportion with *Dbx1*-negative cells), raises doubts about the necessity of the extra *Dbx1*-negative ‘caudoventral’ or ‘ventrolateral caudal’ amygdalar pallial portion conceived theoretically to account for negative halves of nuclei (Puelles et al. 2016a), a notion still recently contemplated by Medina et al. (2017) and Desfilis et al. (2018). The mixture of* Dbx1*-LacZ positive and negative cells in all amygdalar pallial nuclei, including the ‘caudal’ (actually periventricular) ones, is consistent with the fact that the marker seems to be at least *partly present* at *all* the pallial amygdalar progenitor zones at E18.5, as it was at E10.5. This result is further consistent with the alternative interpretation offered in Puelles et al. (2016a) that the pallial ventricular sector producing the *Dbx1*-derived mantle both at cortical and amygdalar levels is apparently accompanied in parallel by a still undefined *Dbx1*-negative neuroepithelial domain that contributes its derivatives to the same amygdalar and cortical formations populated by *Dbx1*-derived progeny (note that also part of the olfactory cortex and the whole olfactory bulb are devoid of *Dbx1*-LacZ-positive cells; Puelles et al. 2016a). The *Dbx1*-derived component predominates at amygdalar levels (as suggested in wholemount preparations in Bielle et al. 2005) and diminishes significantly in a rostralward gradient along cortical VPall levels (a point already underlined by Puelles et al. 2016a). However, at amygdalar levels *varying proportions* of each derived cell population lack the *Dbx1*-LacZ marker. We postulate that this is due to the mixture of similar neurons born at the corresponding *Dbx1*-negative matrix component. The latter may include or correspond to the *Dbx1*-negative ‘ventrocaudal’ amygdalar ventricular portion contributing cells to the MeA of Ruiz-Reig et al. (2018), if it truly belongs to the pallium, as held by these authors (a doubt arises, though, because the MeA is generally assumed to be a part of the subpallium). However, relatively abundant *Dbx1*-positive cells were actually detected by Puelles et al. (2016a) also at the MeA. This perhaps suggests, jointly with the cited data of Ruiz-Reig et al. (2018), that some part of the MeA is perhaps pallial, and accordingly also shows a mixture of *Dbx1*-positive and negative cells.

We conclude from our reappraisal of the *Dbx1* labelling evidence that the whole set of amygdalar pallial derivatives, similarly as the neighboring ventropallial olfactory cortex, are formed from a progenitor domain that combines *Dbx1*-positive and *Dbx1*-negative mother cells. This includes as well the *posterior* radial unit derivatives (AHi/PMCo), which were previously compared (or ascribed) to the MPall (Abellán et al., 2014; Medina et al. 2017).

The *posterior* radial unit /AHi/PMCo) would be the amygdalar portion which is in a position to contact behind the subpallial MeA (we do believe that at least some part of MeA is subpallial) the hypothalamo-amygdalar corridor that extends dorsalward (topologically) the alar hypothalamic paraventricular area, which was detected in *Dbx1*-LacZ material all along the bottom of the terminal sulcus.

### Should the concept of cortical pallial sectors (VPall or others) be applied to amygdalar pallium?

The foregoing argument raises the point whether a *typical* cortical ventropallial subregion (VPall) can be said to extend within the pallial amygdala, just because some gene markers are shared between the structurally very distinct cortical and amygdalar parts of the pallium. We already argued against the idea that amygdalar populations originate from several cortical sectors and migrate separately into the amygdalar complex. This hypothesis was refuted by evidence mentioned above that ventral pallium markers such as *Dbx1* and *Sfrp2* are present independently at both the cortical and amygdalar pallial domains before neurogenesis takes place, and the same applies to markers of the medial pallium such as *Emx2* and *Lhx2*. This makes local production of the respective cortical and amygdalar derivatives parsimonius, and we can forget the non-demonstrated cortico-amygdalar migration streams. Even accepting the available molecular evidence apparently supporting a ‘ventropallial’ similarity of given parts of the pallial amygdala (Medina et al. 2004; Bielle et al. 2005; Teissier et al. 2010; Puelles et al. 2016a), the presently *corrected* combined *Dbx1*/ *Lhx9/ Lhx2/ Emx2/ Tbr1* data in the context of a variety of radial units indicate that, due to its molecular and structural heterogeneity, the hypothetic amygdalar VPall would be *very different* from the homogeneously ‘olfactory’ cortical VPall (Puelles 2014, 2017). Indeed, the *Lhx9*-negative *lateral/basal* units need to be distinguished from the *Lhx9*-positive *anterior* and *posterior* units, apart of between themselves (*lateral* versus *basal*; *basolateral* versus *basomedial*, *basomediolateral* versus *basomediomedial*; Table 1), on the basis of some 80 variously distributed gene patterns (Garcia-Calero et al. 2020). None of this amygdalar molecular complexity is found in the cortical VPall.

It should be noted that the primitive concept of ventral pallium stood originally on a particular apparently homogeneous molecular combination (*Pax6/Dbx1*-positive ventricular zone, plus a *Tbr1*-positive/*Emx1*-negative mantle zone; Smith-Fernández et al. 1998; Puelles et al. 2000, 2016a; Medina et al. 2004; Bielle et al. 2005; Remedios et al. 2004; Tole et al. 2005; García-López et al. 2008; Hirata et al. 2009; Waclaw et al. 2010). The recently discussed *concentric ring model* of the pallium (Puelles et al. 2019) suggests that this initial molecular definition of VPall applies primarily to the *olfactory allocortex* domain placed within the lateral part of the outer cortical ring (see also Puelles 2017). The pallial amygdala is instead a physically separate *nuclear* formation of substantial complexity, which lies topologically *outside* the outer cortical ring. As such it is probably exposed during development to a differential patterning atmosphere of organizing agents (positional signals), insuring intrinsic non-cortical differential pattern and histogenetic fates, which does not preclude the sharing of some developmental transcription factors.

In retrospect, we question the advantages of unifying the olfactory cortical VPall (or any other cortical sector) with the largely different overall structure and singularly varied molecular profile of the nuclearly-structured amygdala (Garcia-Calero et al. 2020). We recently pointed out that, as regards neurogenetic/histogenetic pattern, the cortical pallium is arranged in an inside-out pattern, whereas the amygdalar pallium adopts an outside-in pattern (Garcia-Calero and Puelles 2020). There is no obviously satisfying common causal explanation springing out of such traditional conceptual unification, either for the cortex or the amygdala. Modern causal models of cortical patterning normally leave aside the amygdala (review in Puelles et al. 2019). If we define an amygdalar (non-cortical) part of VPall (or other such sectors), which imply the existence of a particular molecular causal explanation of its adult structure), we immediately require ad hoc added causal explanations to account for the majoritary *differential* nuclear structure of the pallial amygdala (if the causes are the same, how is it that they develop differently?). The amygdala does receive olfactory input (in some of its parts, not all of them), but nevertheless displays important histogenetic variation with respect to the olfactory cortex (this is even more significative when a straightforward *periamygdalar* olfactory cortex is recognized just *outside* of the pallial amygdala, as shown in Garcia-Calero et al. 2020). We do not seem to have such ad hoc explanations at the moment. The same argument applies to DPall with respect to amygdalar NLOT (Remedios et al. 2007), and to hippocampal cortex structure (MPall) relative to the adjoining differently structured *posterior* amygdalar unit (AHi/PMCo; Abellán et al. 2014).

We conclude that it may be simpler to restrict the concepts of VPall, LPall, DPall and MPall to the *cortical pallium*, as conceived either in the updated tetrapartite pallial model (Puelles 2014, 2017), or in the more comprehensive and now preferred concentric ring cortical model (Puelles et al. 2019; note this model adds orbital, cingulate, postrhinal and entorhinal neighbourhoods not contemplated before, and maps as well known secondary organizers). The pallial amygdala obviously can be subdivided in its turn as seems most convenient (either according to present radial units proposed by Garcia-Calero et al. 2020, or by any other strictly amygdalar regionalization system attentive to radial topology, i.e., not based on conventional coronal sections). We do not need to achieve amygdalar consistency with cortical pallial sectors, since surely these adjacent but distinct pallial fields have significantly different patterning mechanisms, given the well-known structural differences. The different overall profile of the amygdalar pallial field compared to the cortical pallial field clearly does not impede that a number of genes cross the mutual boundary in various ways, perhaps also due to some shared patterning effects. These shared signals may even create some shared neuronal properties (e.g., the capacity to receive olfactory projections).

### Is the conventional functional structure of the ‘BM’ amygdala illuminated by the new radial model?

A reviewer objected to our initially purely developmental morphological report, in which we mainly discussed predicted differential causal mechanisms in cortical versus amygdalar parts of the telencephalic pallium. This reviewer alluded to the higher interest of functional analysis for most readers (particularly in a journal whose title comprises ‘Structure and Function’). This assessment is no doubt true, but we think it is premature to construe at this stage from non-functional data a variant functional hypothesis of the amygdalar system. However, in brain science new structural concepts tend sooner or later to suggest novel possibilities of interpretation of available functional data, and we accordingly contribute our modest grain to this enterprise in this final section.

This report centers on the *anterior* amygdalar radial unit, whose novel definition in our model dissociates conceptually the BMA/ACo nuclei showing differential *Lhx9* expression from the classic *Lhx9*-negative BMP nucleus. The latter is thought to derive from the separate *basal* amygdalar radial unit (Garcia-Calero et al. 2020). In contrast, much hodological and functional literature refers indistinctly to ‘the basomedial amygdala’ (‘BM’), a concept that lumps together ACo, BMA, and BMP. We will thus examine whether the correction we suggest illuminates in any way the current schema of functional amygdalar circuitry, as revealed by the connectivity of these three amygdalar centers. We will use as a data source the account of amygdalar connections given recently by Olucha-Bordonau et al. (2015).

ACo receives afferents from the main and accessory olfactory bulbs (Pro-Sistiaga et al. 2007; Gutierrez-Castellanos et al. 2010), the posterior intralaminar thalamic nuclei (non-chemosensory input), the posterior piriform cortex, and insular association areas (processed olfactory input), plus the medial prefrontal cortex (prelimbic and infralimbic areas apparently have roles in gating of fear responses and fear extinction, respectively). These connections are involved in the control of fear behavior, adjusting it to cognitive, contextual, mnemonic and internal state factors (Sotres-Bayon and Quirk 2010). Other connections with the orbitofrontal cortex relate to adjusting outcome expectancies, or emotional responses to unexpected outcomes. Relaxin afferents to ACo from the prepontine nucleus incertus (Olucha-Bordonau et al. 2003; Ryan et al. 2011, 2013) are held to relate this amygdalar centre to anxiety/depression. There are receptors for sexual steroids in the ACo, a feature held to allow integration of chemosensory and endocrine values in the control of socio-sexual behavior (Petrulis 2013). ACo projects contralaterally via the stria terminalis and the anterior commissure, and maintains reciprocal ipsilateral connections with other superficial amygdalar formations, such as the PMCo, PLCo, CxA, NLOT and BAOT (association of olfactory and vomeronasal inputs).

Interestingly, a number of intrinsic amygdalar connections interconnect these superficial centers with the BMA, BLA and La nuclei of the intermediate stratum, as well as with the BLP and BMP nuclei of the periventricular stratum. BMA (jointly with BLA/BLP and AHi) has massive output projections to the extended amygdala and other centers that control behavior, emotion and motivation (e.g., nucleus accumbens, medial amygdala and BST nuclei), and receives feedback from the extended amygdala. These interconnections allow functional interdependence between chemosensory (corticomedial) and non-chemosensory (basolateral) amygdalar divisions, underpinning integrated functional system properties of the amygdala. The amygdalar ‘BM’ outputs targetting the telencephalon (associative perirhinal and insular cortex; stratum lacunosum-moleculare of CA1, as opposed to preferent ‘BL’ outputs to prefrontal cortex and stratum oriens and radiatum of CA3/1). These ‘BM’ outputs are held to be involved in emotional memories and control of attention and motivation. The outputs reaching directly or indirectly the hypothalamus and brainstem control the expression of emotional behavior. The ‘BM’ amygdala participates jointly with the ‘BL’ counterpart in conditioned fear responses, receiving input from the La nucleus and projecting into the subpallial central nucleus (Ce), which spreads its projections to hypothalamus (autonomous response), periaqueductal grey (freezing response), brainstem (startle response) and VSt/DSt (escape response; instrumental learning). Canteras et al. (2001) put forward the hypothesis that ‘BM’ is involved in fear to live predators.

On the whole, we find in this brief course through amygdalar connections and functions related to the *anterior* radial unit studied by us in the present report, that the frequent reference in this literature to the ‘BM’ (sic), as if it were an integrated anatomo-functional complex, is not supported by clear evidence of preferential bidirectional connections between the *Lhx9*-positive BMA/ACo complex and the *Lhx9*-negative BMP nucleus (while BMA and ACo, members of the same radial unit, are indeed strongly interconnected). Recapitulative research is thus needed to discriminate which functions conventionally attributed to the lumped ‘BM’ on the basis of now obsolete anatomic assumptions, belong to each of the distinct ACo/BMA and BMP entities classified by us into different amygdalar radial units. A separate histogenetic origin does not preclude by itself that BMA/ACo collaborates functionally with BMP, but it can be recommended that researchers attend to each of these formations individually, and cease lumping them together automatically into the now anatomically imprecise ‘BM’ category. There is the possibility that such measures clarify the functional significance of these amygdalar components.

## Experimental procedures

### Animal preparation and tissue analysis

The day of the vaginal plug in female mice was counted as embryonic day (E) 0.5. The brains from mouse embryos (E12.5-E18.5) were dissected out and fixed overnight in 4% paraformaldehyde in pH 7.4 phosphate-buffered saline (PBS) at 4 °C. Next, these brains were embedded in 4% agarose in PBS and were sectioned with 100 µm thickness and cut in *amygdalar radial* plane (Garcia-Calero et al. 2020), horizontal, sagittal, and other obliquus planes with a Leica vibratome (VT1000 S) and processed for in situ hybridization and immunohistochemistry. The number of animals used in this work: 3–5 animals for every developmental stage analyzed.

### In situ hybridization

We used restriction enzymes and polymerases in the presence of digoxigenin-11-UTP for riboprobe preparation. Mouse cDNA probes used for in situ hybridization analysis were *Dlx5* (J.R. Rubenstein), *Enc1* (M.C. Hernandez), *Lhx2* and *Lhx9* (our own lab). The hybridization protocol used in the present work was publish in Shimamura et al. (1994).

### Immunohistochemistry

For immunohistochemistry experiments, we followed the protocol published in Garcia-Calero and Scharff (2013). The primary antibodies used in this study were: rabbit anti-calbindin (1:1000; CB-38, Swant, Bellinzona, Switzerland), rabbit anti-Tbr1 (1:200; sc-48816, Santa Cruz Biotechnology, Inc), mouse anti-RC2 (1:10; Developmental Studies Hybridoma Bank, Iowa City, IA).

### Image capture, manipulation and figure assembly

Digital photo micrographs were acquired using Aperio CS2 and processed with Aperio ImageScope (Leica Microsystems GmbH, Mannheim, Germany), Adobe Photoshop and Adobe Illustrator softwares (Adobe Systems MountainView, CA, USA).

## Abbreviations

3v: Third ventricle
AA: Anterior amygdala
ACo: Anterior cortical nucleus
AHi: Amygdalo-hippocampal area
AHiCL: Amygdalo-hippocampal area, caudolateral part
AHiRL: Amygdalo-hippocampal area, rostrolateral part
AHiRM: Amygdalo-hippocampal area, rostromedial part
ant: Anterior radial unit
A. unit: Anterior radial unit
APir: Amygdalo-piriform area
BAOT: Bed nucleus of the accessory olfactory tract
bas: Basal radial unit
BLA: Anterior basolateral nucleus
BLI: Intermediate basolateral nucleus
BLP: Posterior basolateral nucleus
BMA: Anterior basomedial nucleus
BMP: Posterior basomedial nucleus
BMIL: Lateral intermediate basomedial nucleus
BMIM: Medial intermediate basomedial nucleus
BMPL: Lateral posterior basomedial nucleus
BMPM: Medial posterior basomedial nucleus
c: Caudal
CeA: Central amygdalar nucleus
CxA: Cortex-amygdala transitional area
CxAC: Cortex-amygdala transitional area, caudal part
CxAR: Cortex-amygdala transitional area, rostral part
ch: Chorioidal tela
d: Dorsal
DBV: Diagonal band, vertical limb
DBH: Diagonal band, horizontal limb
Ent: Entorhinal cortex
fi: Fimbria of hippocampus
Hi: Hippocampus
l: Lateral
L: Lateral nucleus
lat: Lateral radial unit
LI: Intermediate lateral nucleus
lot: Lateral olfactory tract
lv: Lateral ventricle
m: Medial
MeA: Medial amygdala
MeD: Medial amygdala, dorsal part
MeAV: Medial amygdala, anteroventral part
MeAVc: Medial amygdala, anteroventral part, core
MeAVs: Medial amygdala, anteroventral part, shell
MePV: Medial amygdala, posteroventral part
MePVc: Medial amygdala, posteroventral part, core
MePVs: Medial amygdala, posteroventral part, shell
NLOT: Nucleus of the lateral olfactory tract
ot: Optic tract
OT: Olfactory tuberculum
Pal: Pallium
ped: Peduncle
Pir: Piriform cortex
PLCo: Posterolateral cortical nucleus
PLCoC: Posterolateral cortical nucleus, caudal part
PLCoR: Posterolateral cortical nucleus, rostral part
PMCo: Posteromedial cortical nucleus
PMCoCLi: Posteromedial cortical nucleus, caudolateral part, intermediate layer
PMCoRLi: Posteromedial cortical nucleus, rostrolateral part, intermediate layer
PMCoRMi: Posteromedial cortical nucleus, rostromedial part, intermediate layer
PMCoCLs: Posteromedial cortical nucleus, caudolateral part, superficial layer
PMCoRLs: Posteromedial cortical nucleus, rostrolateral part, superficial layer
PMCoRMs: Posteromedial cortical nucleus, rostromedial part, superficial layer
PThE: Prethalamic eminence
PThEt: Prethalamic eminence, telencephalic part
post: Posterior radial unit
r: Rostral
REP: Retroendopiriform nucleus
REPI: Retroendopiriform intermediate area
REPCo: Retroendopiriform cortical area
SP: Subpallium
Spall: Subpallium
Th: Thalamus
v: Ventral
vpg: Ventropallial glial packet

## References

[CR1] Abellán A, Legaz I, Vernier B, Rétaux S, Medina L (2009). Olfactory and amygdalar structures of the chicken ventral pallium based on the combinatorial expression patterns of LIM and other developmental regulatory genes. J Comp Neurol.

[CR2] Abellán A, Vernier B, Rétaux S, Medina L (2010). Similarities and differences in the forebrain expression of Lhx1 and Lhx5 between chicken and mouse: Insights for understanding telencephalic development and evolution. J Comp Neurol.

[CR3] Abellán A, Desfilis E, Medina L (2013). The olfactory amygdala in amniotes: an evo-devo approach. Anat Rec (Hoboken).

[CR4] Abellán A, Desfilis E, Medina L (2014). Combinatorial expression of *Lef1*, *Lhx2*, *Lhx5*, *Lhx9*, *Lmo3*, *Lmo4*, and *Prox1* helps to identify comparable subdivisions in the developing hippocampal formation of mouse and chicken. Front Neuroanat.

[CR5] Alheid GF, de Olmos J, Beltramino CA, Paxinos G (1995). Amygdala and extended amygdala. The rat nervous system.

[CR6] Alonso A, Trujillo CM, Puelles L (2020). Longitudinal developmental analysis of prethalamic eminence derivatives in the chick by mapping of Tbr1 in situ expression. Brain Struct Funct.

[CR7] Amaral DG, Bauman MD, Schumann CM (2003). The amygdala and autism: implications from nonhuman primate studies. Genes Brain Behav.

[CR8] Bachy I, Vernier P, Retaux S (2001). The LIM-homeodomain gene family in the developing Xenopus brain: conservation and divergences with the mouse related to the evolution of the forebrain. J Neurosci.

[CR9] Bertuzzi S, Porter FD, Pitts A, Kumar M, Agulnick A, Wassif C, Westphal H (1999). Characterization of Lhx9, a novel LIM/homeobox gene expressed by the pioneer neurons in the mouse cerebral cortex. Mech Dev.

[CR10] Bielle F, Griveau A, Narboux-Nême N, Vigneau S, Sigrist M, Arber S, Wassef M, Pierani A (2005). Multiple origins of Cajal-Retzius cells at the borders of the developing pallium. Nat Neurosci.

[CR11] Bulchand S, Subramanian L, Tole S (2003). Dynamic spatiotemporal expression of LIM genes and cofactors in the embryonic and postnatal cerebral cortex. Dev Dyn.

[CR12] Bupesh M, Abellán A, Medina L (2011). Genetic and experimental evidence supports the continuum of the central extended amygdala and a mutiple embryonic origin of its principal neurons. J Comp Neurol.

[CR13] Burdach KF (1819–1822) Vom Baue und Leben des Gehirns, Leipzig

[CR14] Canteras NS, Ribeiro-Barbosa ER, Comoli E (2001). Tracing from the dorsal premammillary nucleus prosencephalic systems involved in the organization of innate fear responses. Neurosci Biobehav Rev.

[CR15] Carney RS, Mangin JM, Hayes L, Mansfield K, Sousa VH, Fishell G, Machold RP, Ahn S, Gallo V, Corbin JG (2010). Sonic hedgehog expressing and responding cells generate neuronal diversity in the medial amygdala. Neural Dev.

[CR16] Choi GB, Dong HW, Murphy AJ, Valenzuela DM, Yancopoulos GD, Swanson LW, Anderson DJ (2005). *Lhx6* delineates a pathway mediating innate reproductive behaviors from the amygdala to the hypothalamus. Neuron.

[CR17] Cobos I, Long JE, Thwin MT, Rubenstein JL (2006). Cellular patterns of transcription factor expression in developing cortical interneurons. Cereb Cortex.

[CR18] Cocas LA, Georgala PA, Mangin JM, Clegg JM, Kessaris N, Haydar TF, Gallo V, Price DJ, Corbin JG (2011). Pax6 is required at the telencephalic pallial-subpallial boundary for the generation of neuronal diversity in the postnatal limbic system. J Neurosci.

[CR19] De Olmos J, Alheid GF, Beltramino C, Paxinos G (1985). Amygdala. The rat nervous system.

[CR20] De Olmos JS, Beltramino CA, Alheid G, Paxinos G (2004). Amygdala and extended amygdala of the rat: a cytoarchitectonical, fibroarchitectonical, and chemoarchitectonical survey. The rat nervous system.

[CR21] Desfilis E, Abellán A, Sentandreu V, Medina L (2018). Expression of regulatory genes in the embryonic brain of a lizard and implications for understanding pallial organization and evolution. J Comp Neurol.

[CR22] Deussing J, Wurst W (2007). Amygdala and neocortex: common origins and shared mechanisms. Nat Neurosci.

[CR23] Fan CM, Kuwana E, Bulfone A, Fletcher CF, Copeland NG, Jenkins NA, Crews S, Martinez S, Puelles L, Rubenstein JL, Tessier-Lavigne M (1996). Expression patterns of two murine homologs of Drosophila single-minded suggest possible roles in embryonic patterning and in the pathogenesis of Down syndrome. Mol Cell Neurosci.

[CR24] Garcia-Calero E (2005) Contribuciones a la regionalizacion telencefalica. Doctoral dissertation, University of Murcia, Spain

[CR27] García-Calero E, Puelles L (2005) Pallial expression of* Enc1* RNA in postnatal mouse telencephalon. Brain Res Bull 66:445–448 10.1016/j.brainresbull.2005.05.003 16144629

[CR100] Garcia-Calero E, Puelles L (2020) Histogenetic Radial Models as Aids to Understanding Complex Brain Structures: The Amygdalar Radial Model as a Recent Example. Front Neuroanat 14:590011. 10.3389/fnana.2020.590011 10.3389/fnana.2020.590011 PMC7683391 33240050

[CR25] Garcia-Calero E, Scharf C (2013). Calbindin expression in developing striatum of zebra inches and its relation to the formation of area X. J Comp Neurol.

[CR26] Garcia-Calero E, Martínez-de-la-Torre M, Puelles L (2020) A radial histogenetic model of the mouse pallial amygdala. Brain Struct Funct. 10.1007/s00429-020-02097-4 10.1007/s00429-020-02097-4 PMC7473974 32583144

[CR200] Garcia-Calero E, López-González L, Martínez-de-la-Torre M, Fan CM, Puelles L (2021) *Sim1*-expressing cells illuminate the origin and course of migration of the nucleus of the lateral olfactory tract in the mouse amygdala. Brain Struct Funct. 10.1007/s00429-020-02197-1 10.1007/s00429-020-02197-1 PMC7910384 33492553

[CR28] García-López M, Abellán A, Legaz I, Rubenstein JL, Puelles L, Medina L (2008). Histogenetic compartments of the mouse centromedial and extended amygdala based on gene expression patterns during development. J Comp Neurol.

[CR102] García-Moreno F, Pedraza M, Di Giovannantonio LG, Di Salvio M, López-Mascaraque L, Simeone A, De Carlos JA (2010) A neuronal migratory pathway crossing from diencephalon to telencephalon populates amygdala nuclei. Nat Neurosci 13:680-689. 10.1038/nn.2556 10.1038/nn.2556 20495559

[CR29] Gorski JA, Talley T, Qiu M, Puelles L, Rubenstein JL, Jones KR (2002). Cortical excitatory neurons and glia, but not GABAergic neurons, are produced in the Emx1-expressing lineage. J Neurosci.

[CR30] Gutierrez-Castellanos N, Martinez-Marcos A, Martínez-García F, Lanuza E (2010). Chemosensory function of the amygdala. Vitam Horm.

[CR31] Hevner RF, Shi L, Justice N, Hsueh Y, Sheng M, Smiga S, Bulfone A, Goffinet AM, Campagnoni AT, Rubenstein JL (2001). Tbr1 regulates differentiation of the preplate and layer 6. Neuron.

[CR103] Hirata T, Li P, Lanuza GM, Cocas LA, Huntsman MM, Corbin JG (2009). Identification of distinct telencephalic progenitor pools for neuronal diversity in the amygdala. Nat Neurosci.

[CR33] Holmgren N (1925). Points of view concerning forebrain morphology in higher vertebrates. Acta Zool.

[CR34] Huilgol D, Udin S, Shimogori T, Saha B, Roy A, Aizawa S, Hevner RF, Meyer G, Ohshima T, Pleasure SJ, Zhao Y, Tole S (2013). Dual origins of the mammalian accessory olfactory bulb revealed by an evolutionarily conserved migratory stream. Nat Neurosci.

[CR35] Johnston JB (1923). Further contributions to the study of the evolution of the forebrain. J Comp Neurol.

[CR36] Kim AS, Anderson SA, Rubenstein JRL, Lowenstein DH, Pleasure SJ (2001). Pax-6 regulates expression of SFRP-2 and Wnt-7b in the developing CNS. J. Neurosci..

[CR37] Krettek JE, Price JL (1978). A description of the amygdaloid complex in the rat and cat with observations on intra-amygdaloid axonal connections. J Comp Neurol.

[CR150] Kuhlenbeck H (1924) Über die Homologien der Zellmassen im Hemi= sphärenhirn der Wirbeltiere. Folia Anatomica Japonica

[CR104] Kuhlenbeck H (1927) Vorlesungen über das Zentralnervensystem der Wirbeltiere. Jena: Fischer

[CR38] Kuhlenbeck H (1973). The central nervous system of vertebrates. 3, Part II: overall morphological pattern.

[CR39] LeDoux J (2007). The amygdala. Curr Biol.

[CR40] Lischinsky JE, Sokolowski K, Li P, Esumi S, Kamal Y, Goodrich M, Oboti L, Hammond TR, Krishnamoorthy M, Feldman D, Huntsman M, Liu J, Corbin JG (2017). Embryonic transcription factor expression in mice predicts medial amygdala neuronal identity and sex-specific responses to innate behavioral cues. Elife.

[CR41] Loo YT (1930). The forebrain of the opossum, *Didelphis virginiana*: I. Gross morphology. J Comp Neurol.

[CR42] Loo YT (1931). The forebrain of the opossum, *Didelphis virginiana*: II. Histology. J Comp Neurol.

[CR43] Marín O, Rubenstein JL (2001). A long, remarkable journey: tangential migration in the telencephalon. Nat Rev Neurosci.

[CR44] Martínez-García F, Novejarque A, Lanuza E (2008). Two interconnected functional systems in the amygdala of amniote vertebrates. Brain Res Bull.

[CR45] Martínez-García F, Noverjarque A, Guitiérrez-Castellanos N, Lanuza E, Watson C, Paxinos G, Puelles L (2012). Piriform cortex and amygdala. The mouse nervous system.

[CR46] Medina L, Legaz I, González G, de Castro F, Rubenstein JLR, Puelles L (2004). Expression of Dbx1, neurogenin 2, semaphorin 5A, cadherin 8, and emx1 distinguish ventraland lateral pallial histogenetic divisions in the developing claustroamygdaloid complex. J Comp Neurol.

[CR47] Medina L, Abellán A, Vicario A, Castro-Robles B, Desfilis E, Kaas J (2017). The Amygdala. Evolution of nervous systems 2e.

[CR48] Moreno N, Bachy I, Rétaux S, González A (2004). LIM-homeodomain genes as developmental and adult genetic markers of Xenopus forebrain functional subdivisions. J Comp Neurol.

[CR49] Nakagawa Y, O'Leary DD (2001). Combinatorial expression patterns of LIM-homeodomain and other regulatory genes parcellate developing thalamus. J Neurosci.

[CR50] Nieuwenhuys R, Puelles L (2016). Towards a new neuromorphology.

[CR51] Olucha-Bordonau FE, Teruel V, Barcia-Gonzalez J, Ruiz-Torner A, Valverde-Navarro AA, Martinez-Soriano F (2003). Cytoarchitecture and efferent projections of the nucleus incertus of the rat. J Comp Neurol.

[CR52] Olucha-Bordonau FE, Fortes-Marco L, Otero-García M, Lanuza E, Martínez-García F (2015). Amygdala: structure and function. The rat nervous system.

[CR53] Petrulis A (2013). Chemosignals, hormones and mammalian reproduction. Horm Behav.

[CR54] Phelps EA, LeDoux JE (2005). Contributions of the amygdala to emotion processing: from animal models to human behavior. Neuron.

[CR55] Pitkänen A, Savander V, LeDoux JE (1997). Organization of intra-amygdaloid circuitries in the rat: an emerging framework for understanding functions of the amygdala. Trends Neurosci.

[CR56] Pro-Sistiaga P, Mohedano-Moriano A, Ubeda-Banon I, Del Mar A-J, Marcos P, Artacho-Perula E, Crespo C, Insausti R, Martinez-Marcos A (2007). Convergence of olfactory and vomeronasal projections in the rat basal telencephalon. J Comp Neurol.

[CR57] Puelles L (2014) Development and evolution of the claustrum. In: The Claustrum. Elsevier

[CR58] Puelles L (2017). Comments on the updated tetrapartite pallium model in the mouse and chick, featuring a homologous claustro-insular complex. Brain Behav Evol.

[CR60] Puelles L (2019). Survey of midbrain, diencephalon, and hypothalamus neuroanatomic terms whose prosomeric definition conflicts with columnar tradition. Front Neuroanat.

[CR61] Puelles L, Rubenstein JL (2003). Forebrain gene expression domains and the evolving prosomeric model. Trends Neurosci.

[CR62] Puelles L, Rubenstein JLR (2015). A new scenario of hypothalamic organization: rationale of new hypotheses introduced in the updated prosomeric model. Front Neuroanat.

[CR63] Puelles L, Kuwana E, Puelles E, Bulfone A, Shimamura K, Keleher J, Smiga S, Rubenstein JLR (2000). Pallial and subpallial derivatives in the embryonic chick and mouse telencephalon, traced by the expression of the genes *Dlx-2*, *Emx-1*, *Nkx-2.1*, *Pax-6*, and *Tbr-1*. J Comp Neurol.

[CR65] Puelles L, Harrison M, Paxinos G, Watson C (2013). A developmental ontology for the mammalian brain based on the prosomeric model. Trends Neurosci.

[CR67] Puelles L, Medina L, Borello U, Legaz I, Teissier A, Pierani A, Rubenstein JLR (2016). Radial derivatives of the mouse ventral pallium traced with Dbx1-LacZ reporters. J Chem Neuroanat.

[CR66] Puelles L, Ayad A, Alonso A, Sandoval JE, MartÍnez-de-la-Torre M, Medina L, Ferran JL (2016). Selective early expression of the orphan nuclear receptor Nr4a2 identifies the claustrum homolog in the avian mesopallium: impact on sauropsidian/mammalian pallium comparisons. J Comp Neurol.

[CR68] Puelles L, Alonso A, García-Calero E, Martínez-de-la-Torre M (2019). Concentric ring topology of mammalian cortical sectors and relevance for patterning studies. J Comp Neurol.

[CR101] Puelles L, Diaz C, Stühmer T, Ferran JL, Martínez-de la Torre M, Rubenstein JLR (2020) LacZ-reporter mapping of Dlx5/6 expression and genoarchitectural analysis of the postnatal mouse prethalamus. J Comp Neurol. 10.1002/cne.24952 10.1002/cne.24952 PMC7671952 32420617

[CR69] Remedios R, Subramanian L, Tole S (2004). LIM genes parcellate the embryonic amygdala and regulate its development. J Neurosci.

[CR105] Remedios R, Huilgol D, Saha B, Hari P, Bhatnagar L, Kowalczyk T, Hevner RF, Suda Y, Aizawa S, Ohshima T, Stoykova A, Tole S (2007). A stream of cells migrating from the caudal telencephalon reveals a link between the amygdala and neocortex. Nature Neurosci.

[CR70] Rétaux S, Rogard M, Bach I, Failli V, Besson MJ (1999). Lhx9: a novel LIM-homeodomain gene expressed in the developing forebrain. J Neurosci.

[CR71] Rolls ET (2014). Emotion and decision-making explained.

[CR72] Rolls ET (2015). Limbic systems for emotion and for memory, but no single limbic system. Cortex.

[CR73] Ruiz-Reig N, Studer M (2017). Rostro-Caudal and Caudo-Rostral Migrations in the Telencephalon: Going Forward or Backward?. Front Neurosci.

[CR74] Ruiz-Reig N, Andrés B, Huilgol D, Grove EA, Tissir F, Tole S, Theil T, Herrera E, Fairén A (2017). Lateral thalamic eminence: a novel origin for mGluR1/Lot cells. Cereb Cortex.

[CR75] Ruiz-Reig N, Andres B, Lamonerie T, Theil T, Fairén A, Studer M (2018). The caudo-ventral pallium is a novel pallial domain expressing Gdf10 and generating Ebf3-positive neurons of the medial amygdala. Brain Struct Funct.

[CR76] Ryan PJ, Ma S, Olucha-Bordonau FE, Gundlach AL (2011). Nucleus incertus—an emerging modulatory role in arousal, stress and memory. Neurosci Biobehav Rev.

[CR77] Ryan PJ, Buchler E, Shabanpoor F, Hossain MA, Wade JD, Lawrence AJ, Gundlach AL (2013). Central relaxin-3 receptor (RXFP3) activation decreases anxiety- and depressive-like behaviours in the rat. Behav Brain Res.

[CR78] Shimamura K, Hirano S, McMahon AP, Takeichi M (1994). *Wnt-1*-dependent regulation of local E-cadherin and alpha N-catenin expression in the embryonic mouse brain. Development.

[CR79] Shirasaki R, Pfaff SL (2002). Transcriptional codes and the control of neuronal identity. Annu Rev Neurosci.

[CR80] Smith-Fernandez A, Pieau C, Reperant J, Boncinelli E, Wassef M (1998). Expression of the *Emx-1* and *Dlx-1* homeobox genes define three molecularly distinct domains in the telencephalon of mouse, chick, turtle and frog embryos: implications for the evolution of telencephalic subdivisions in amniotes. Development.

[CR81] Sotres-Bayon F, Quirk GJ (2010). Prefrontal control of fear: more than just extinction. Curr Opin Neurobiol.

[CR82] Stenman JM, Wang B, Campbell K (2003). Tlx controls proliferation and patterning of lateral telencephalic progenitor domains. J Neurosci.

[CR106] Striedter GF (1997). The telencephalon of tetrapods in evolution. Brain Behav Evol.

[CR84] Swanson LW, Petrovich GD (1998). What is the amygdala?. Trends Neurosci.

[CR85] Teissier A, Griveau A, Vigier L, Piolot T, Borello U, Pierani A (2010). A novel transient glutamatergic population migrating from the pallial-subpallial boundary contributes to neocortical development. J Neurosci.

[CR86] Tole S, Remedios R, Saha B, Stoykova A (2005). Selective requirement of *Pax6*, but not Emx2, in the specification and development of several nuclei of the amygdaloid complex. J Neurosci.

[CR87] Tsuchida T, Ensini M, Morton SB, Baldassare M, Edlund T, Jessell TM, Pfaff SL (1994). Topographic organization of embryonic motor neurons defined by expression of LIM homeobox genes. Cell.

[CR88] Waclaw RR, Ehrman LA, Pierani A, Campbell K (2010). Developmental origin of the neuronal subtypes that comprise the amygdalar fear circuit in the mouse. J Neurosci.

[CR89] Weiskrantz L (1956). Behavioral changes associated with ablation of the amygdaloid complex in monkeys. J Comp Physiol Psychol.

[CR90] Yun K, Potter S, Rubenstein JL (2001). Gsh2 and Pax6 play complementary roles in dorsoventral patterning of the mammalian telencephalon. Development.

